# Cardiac troponin T and 28-day mortality in traumatic brain injury: a retrospective cohort study and machine learning prediction model

**DOI:** 10.3389/fneur.2026.1926632

**Published:** 2026-10-07

**Authors:** Liwen Zhang, Wenwen Hu, Wenjie He, Feiyu Huang, Jing Zhang

**Affiliations:** Department of Neurocritical Care, Taihe Hospital, Hubei University of Medicine, Shiyan, Hubei, China

**Keywords:** 28-day mortality, cardiac troponin T, machine learning, MIMIC database, random forest, traumatic brain injury

## Abstract

**Background:**

Traumatic brain injury (TBI) frequently induces cardiac dysfunction via the brain-heart axis, yet the prognostic value of cardiac troponin T (cTnT) in TBI remains incompletely characterized. This study investigated thye association between cTnT levels and 28-day all-cause mortality in TBI patients and developed a machine learning-based prediction model.

**Methods:**

This retrospective cohort study included 837 adult TBI patients from the MIMIC-III/IV databases (development cohort) and 121 patients from a single Chinese center (external validation cohort). The first cTnT measurement within 24 h of ICU admission was analyzed. Restricted cubic spline (RCS) regression was used to explore nonlinear relationships. cTnT was categorized into quartiles (Q1 ≤ 0.03, Q2 0.03–0.06, Q3 0.06–0.17, Q4 > 0.17 ng/mL). A two-step Boruta plus LASSO feature selection identified 13 robust predictors. Five machine learning algorithms (Random Forest, BART, GBM, CTREE, SVM) were trained. The primary outcome was 28-day all-cause mortality.

**Results:**

Among 837 patients, 217 (25.9%) died within 28 days. RCS revealed a significant nonlinear association (P for nonlinearity < 0.001). In multivariable Cox regression adjusted for the 12 LASSO-selected covariates (excluding cTnT quartile itself), patients in Q3 and Q4 had nearly twofold higher mortality risks compared with Q1 (HR = 2.01, 95% CI: 1.37–2.95, *p* < 0.001; and HR = 1.96, 95% CI: 1.31–2.93, *p* = 0.001, respectively). Random Forest achieved the best discriminative performance, with an internal AUC of 0.783 and an external validation AUC of 0.706 (95% CI: 0.584–0.828). The model also demonstrated good calibration (Brier score = 0.155) and favorable clinical utility across clinically relevant threshold probabilities as shown by decision curve analysis.

**Conclusion:**

Elevated cTnT is independently associated with 28-day mortality in TBI patients, with a potential risk threshold of ≥0.06 ng/mL. The Random Forest model integrating cTnT with clinical variables provides moderate mortality prediction. cTnT may serve as an integrative marker of systemic stress and multi-organ dysfunction in TBI.

## Introduction

Traumatic brain injury (TBI) is one of the leading causes of death and disability worldwide (1–3). Its complex pathophysiological mechanisms affect not only the central nervous system but also trigger systemic inflammatory responses and multi-organ dysfunction (4–6). In recent years, experimental and clinical studies have increasingly revealed an “invisible” interaction between the heart and brain. This interaction is particularly evident in TBI: even in the absence of pre-existing cardiac disease, TBI can induce cardiac dysfunction (7), thereby increasing mortality risk and potentially leading to long-term cardiac complications such as heart failure (8) and neurogenic stress cardiomyopathy (NSC) (9–11).

The pathophysiological mechanisms underlying cardiac dysfunction after TBI are highly complex, involving enhanced sympathetic nervous activity, activation of the hypothalamic–pituitary–adrenal (HPA) axis, and dysregulation of immune and inflammatory pathways. Together, these processes contribute to autonomic dysfunction, excessive catecholamine release, cardiomyocyte injury, mitochondrial impairment, and sustained inflammatory cascades (12–14). Although substantial progress has been made in understanding the brain–heart axis in stroke (15, 16) and subarachnoid hemorrhage (SAH) (17), research specifically addressing the brain-heart axis in TBI patients remains limited (18).

Cardiac troponin T (cTnT) is a highly specific and sensitive biomarker traditionally used for diagnosing myocardial injury and acute coronary syndromes (19–21). However, growing evidence suggests that elevated cTnT levels are not only associated with cardiac events but also reflect systemic inflammatory responses and multi-organ dysfunction (22, 23). Mechanistically, cTnT serves as a direct marker of myocardial injury and may also indicate the presence of systemic inflammatory response syndrome (SIRS) and multiple organ dysfunction syndrome (MODS) following TBI (15). After TBI, systemic inflammation is initiated, leading to cardiomyocyte damage and subsequent release of troponin into the circulation. Thus, changes in cTnT levels represent not only cardiac injury but also serve as an important indicator of systemic inflammatory status.

Against this backdrop, this study aims to investigate the association between cTnT levels and clinical outcomes in TBI patients and to evaluate its prognostic value. We utilized data from large critical care databases (Medical Information Mart for Intensive Care III and IV) and employed multiple machine learning approaches to develop survival prediction models for TBI patients, with the goal of improving prediction accuracy and reliability. Additionally, we applied restricted cubic spline (RCS) analysis to explore the potential nonlinear relationship between cTnT and patient outcomes. These findings are expected to provide valuable insights for clinical decision-making in TBI management and lay the groundwork for further investigation into the complex determinants of prognosis (24, 25).

## Methods

### Data sources

This study employed a retrospective observational design. Data were sourced from two components: a public database for model development and internal validation, and an independent single-center dataset for external validation.

### Model development cohort

Data were integrated from the Medical Information Mart for Intensive Care III (MIMIC-III, v1.4) and MIMIC-IV (v3.1) databases. To ensure patient data independence, for individuals present in both databases during the overlapping period (2008–2012), only their MIMIC-IV records were retained. These databases contain detailed clinical information, including vital signs, laboratory test results, medication records, diagnoses, and treatment procedures. All data were rigorously de-identified in compliance with privacy protection regulations and received a waiver of informed consent and approval for data sharing from the Institutional Review Board (IRB) of Beth Israel Deaconess Medical Center (BIDMC) (26). The author (Liwen Zhang) holds authorized access (certification number: 66943022).

MIMIC-III includes data from adult patients admitted to ICUs at Beth Israel Deaconess Medical Center in Boston, USA, from 2001 to 2012.

MIMIC-IV, its updated successor, covers data from 2008 to 2019.

### External validation cohort

To assess model generalizability, we retrospectively collected data from all eligible traumatic brain injury (TBI) patients admitted to the Department of Neurocritical Care at Taihe Hospital, Shiyan City, Hubei Province, China, between January 31, 2023, and October 31, 2023. This study protocol was reviewed and approved by the Ethics Committee of Taihe Hospital (Approval No. 2025KS181) in accordance with the Declaration of Helsinki and the Measures for Ethical Review of Biomedical Research Involving Human Subjects (2016). Among 135 screened patients, 121 met the inclusion and exclusion criteria and were included in the external validation set. The external validation dataset used for machine learning analysis consisted exclusively of patients fulfilling all predefined eligibility criteria.

### Biomarker measurement

Development cohort (MIMIC-III/IV): cTnT values were extracted from the databases as recorded. The MIMIC databases do not uniformly specify the assay platform; therefore, assay-related differences between cohorts should be considered when interpreting external validation results (27).

External validation cohort (Taihe Hospital, China): cTnT was measured using the ReLIA Cardiac Troponin T assay kit (immunofluorescence method, ReLIA Biotechnology, Jiangsu, China), according to the manufacturer’s instructions. The assay employs a sandwich immunofluorescence principle. The lower limit of detection (LoD) is 0.01 ng/mL, with a 95th percentile reference limit of 0.04 ng/mL. The assay is traceable to the U.S. National Institute of Standards and Technology (NIST) Standard Reference Material (SRM2921). All measurements were performed on the ReLIA immunofluorescence analyzer within 24 h of ICU admission.

### Inclusion and exclusion criteria

Inclusion Criteria (all must be satisfied):

Adult patients: age ≥ 18 years.

Confirmed diagnosis: traumatic brain injury (TBI) identified by International Classification of Diseases, Ninth or Tenth Revision (ICD-9 or ICD-10) clinical diagnosis codes.

First ICU admission: the patient’s first admission to the intensive care unit during the current hospitalization.

Exclusion Criteria (any one leads to exclusion):

Missing core variable: no cardiac troponin T (cTnT) measurement within 24 h of ICU admission.

Missing key outcome: 28-day post-admission survival status undetermined (lost to follow-up).

Competing risk of mortality: documented history of myocardial infarction prior to hospital admission.

Data duplication or poor quality: for patients appearing in both MIMIC-III and MIMIC-IV, only MIMIC-IV records were retained.

### Core variables and outcomes

Core exposure variable: cardiac troponin T (cTnT), defined as the first plasma concentration (unit: ng/mL) measured within 24 h of ICU admission.

Primary outcome: all-cause mortality at 28 days after hospital admission.

Secondary outcome: in-hospital all-cause mortality.

### Statistical analysis

#### Data extraction

Patient-level data were extracted from the databases, including demographic characteristics (e.g., age, body weight), vital signs (heart rate, respiratory rate, systolic blood pressure, diastolic blood pressure, and mean arterial pressure), and laboratory parameters (glucose, hemoglobin, platelet count, white blood cell count, cardiac troponin T, alanine aminotransferase, aspartate aminotransferase, blood urea nitrogen, creatinine, total bilirubin, total calcium, chloride, potassium, sodium, bicarbonate, lactate, partial pressure of carbon dioxide, and partial pressure of oxygen). Comorbidities—including hypertension, ischemic stroke, type 2 diabetes, hyperlipidemia, and heart failure—were identified using International Classification of Diseases, Ninth and Tenth Revision (ICD-9 and ICD-10) diagnosis codes. Therapeutic interventions, specifically vasopressor use (binary: yes/no), were extracted from medication records. Additionally, the Glasgow Coma Scale (GCS) score (28, 29), length of hospital stay, and vital status at 28 days were recorded for all patients.

#### Data preprocessing

All statistical analyses were conducted using DecisionLinnc (Statsape Co., Ltd., Hangzhou, China) (30) and R software (version 4.4.2). The use of these platforms does not alter the underlying analytical algorithms, and all results can be fully reproduced using standard code.

Normality of continuous variables was assessed using the Kolmogorov–Smirnov test. Normally distributed variables are presented as mean ± standard deviation and compared between two groups using the independent-samples t-test; non-normally distributed variables are reported as median (interquartile range) and compared using the Mann–Whitney U test. Nonparametric methods were applied for the majority of variables due to the skewed distributions commonly observed in ICU-related laboratory parameters. For comparisons involving three or more groups, the Kruskal–Wallis test was used for continuous variables.

For all other predictor variables, missingness was below the pre-specified 40% exclusion threshold (31–33). This threshold was chosen based on established recommendations for critical care EHR data, as variables with missing rates exceeding 40% often exhibit non-random missing patterns that may introduce bias when imputed, particularly in ICU electronic health record data (33). Missing values were imputed using the k-nearest neighbors (k-NN) algorithm, a non-parametric method widely used for missing data imputation in healthcare datasets (34). The missing proportion for each variable is provided in Supplementary Table S1. The k-NN imputation was performed with k = 5 (selected by cross-validation). Patients with undetermined 28-day survival status were excluded from the analysis, as specified in the eligibility criteria. All statistical tests were two-sided, and *p* < 0.05 was considered statistically significant.

#### Association between cTnT and 28-day mortality risk

Restricted cubic spline (RCS) regression was performed to explore the potential nonlinear association between cTnT (modeled as a continuous variable) and 28-day all-cause mortality risk, with three knots placed at 0.01, 0.04, and 0.11 ng/mL (selected based on the distribution of cTnT).

### Prediction model development and validation

#### Variable selection

To minimize overfitting and avoid the bias inherent in univariate pre-screening, a two-stage feature selection strategy was employed. First, the Boruta algorithm—a random forest-based wrapper method that iteratively compares the importance of each predictor against randomly permuted “shadow” attributes—was applied to all 31 candidate variables in the development cohort. Variables classified as “Confirmed” by Boruta were retained for subsequent analysis. Second, the retained variables were entered into a least absolute shrinkage and selection operator (LASSO) regression with 10-fold cross-validation. The penalty parameter λ was selected using the λ.min criterion, which minimizes the cross-validated partial likelihood deviance. Variables with non-zero coefficients at λ.min were retained as the final predictor set. LASSO identified 13 predictors including cTnT quartile. In multivariable Cox regression models, cTnT quartile was the exposure of interest, and the remaining 12 variables were included as covariates. Machine learning models (Random Forest, GBM, BART, CTREE, and SVM) used all 13 variables. This final predictor set was used consistently across all multivariable Cox regression and machine learning models.

#### Multivariable cox regression

The LASSO-selected variables were entered into a multivariable Cox proportional hazards regression model to evaluate the independent prognostic value of cTnT after adjusting for potential confounders.

#### Machine learning model development

The development cohort was randomly split into a training set (*n* = 586) and an internal test set (*n* = 251) in a 7:3 ratio. Using the same LASSO-selected feature set, five machine learning algorithms—Random Forest, Gradient Boosting Machine (GBM), Bayesian Additive Regression Trees (BART), Conditional Inference Tree (CTREE), and Support Vector Machine (SVM)—were trained on the training set with 5-fold cross-validation.

#### Model evaluation

Discriminative performance was assessed using the area under the receiver operating characteristic curve (AUC). The best-performing model was evaluated on both the internal test set and the external validation cohort (*n* = 121). To address potential sources of bias, multivariable adjustment was used to control for confounding, and external validation was performed to reduce overfitting and assess model generalizability.

Model calibration was assessed using calibration curves and the Brier score. Clinical utility was evaluated using decision curve analysis (DCA).

## Results

### Baseline characteristics

This study included a total of 837 adult patients with traumatic brain injury (TBI) admitted to the intensive care unit (ICU), including 280 from the MIMIC-III database and 557 from the MIMIC-IV database. The primary outcome was 28-day all-cause mortality (Figure 1). Baseline characteristics of patients stratified by 28-day outcome—survivors (*n* = 620, 74.1%) and non-survivors (*n* = 217, 25.9%)—are presented in Table 1.

**Figure 1:**
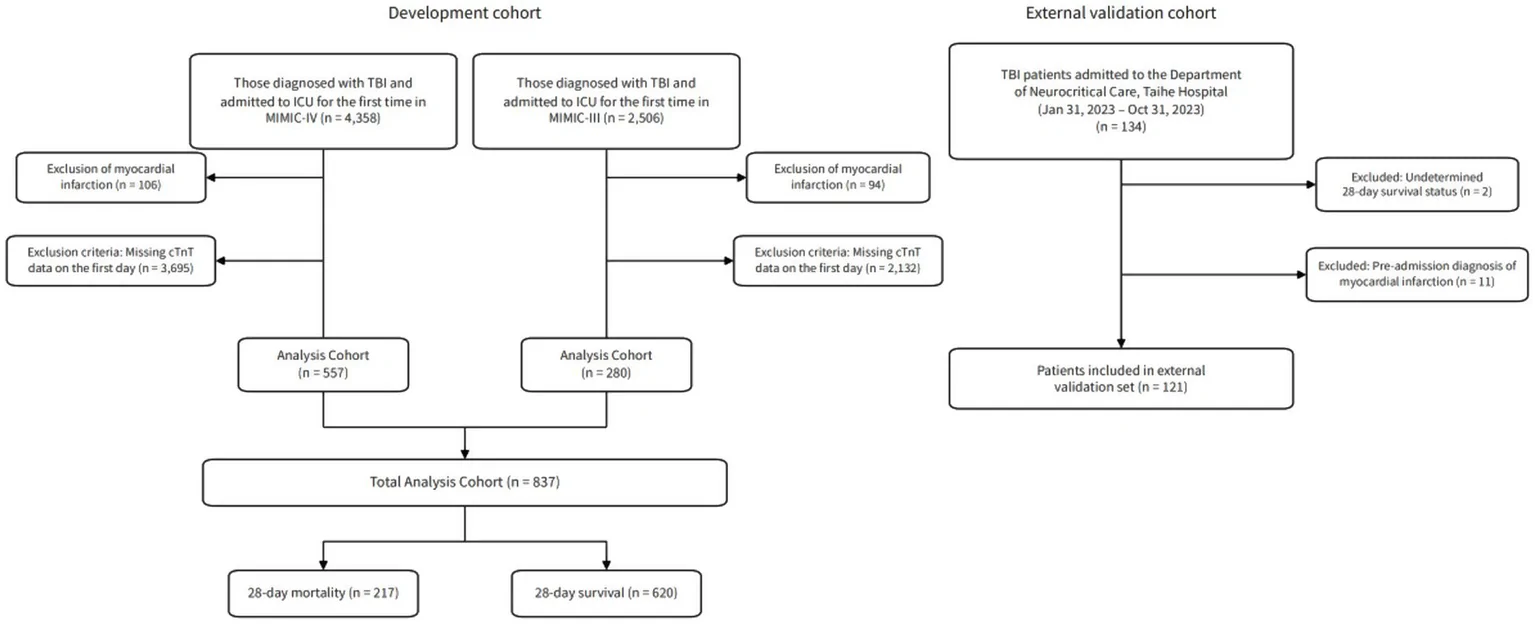
Patient selection flowchart.

**Table 1:** Baseline characteristics of traumatic brain injury patients stratified by 28-day mortality status.

Variable	Survivors (*n* = 620)	Non-survivors (*n* = 217)	*p*-value
Demographics			
Age, years	75.00 (61.00–84.00)	78.00 (63.00–87.00)	0.048
Female sex, *n* (%)	221 (35.65%)	82 (37.79%)	0.572
Vital signs			
Heart rate, beats/min	90.50 (77.00–100.95)	93.00 (82.00–105.20)	0.009
Systolic blood pressure, mmHg	132.00 (116.00–146.00)	128.70 (112.00–144.00)	0.090
Diastolic blood pressure, mmHg	71.00 (61.40–80.00)	70.80 (61.00–78.60)	0.578
Mean arterial pressure, mmHg	86.00 (77.00–97.00)	85.00 (76.00–94.00)	0.286
Respiratory rate, breaths/min	19.00 (16.00–22.00)	19.90 (16.00–22.00)	0.103
Neurological status			
Glasgow Coma Scale score	14.00 (13.00–15.00)	15.00 (11.00–15.00)	0.526
Hematology			
Hemoglobin, g/dL	10.70 (9.30–12.20)	10.70 (9.40–12.60)	0.438
Platelet count, ×10^9^/L	182.50 (140.00–236.00)	180.00 (129.00–239.00)	0.348
White blood cell count, ×10^9^/L	9.75 (7.30–13.45)	12.50 (9.40–17.20)	<0.001
Electrolytes			
Calcium, mg/dL	8.50 (7.90–9.00)	8.30 (7.60–9.00)	0.103
Potassium, mmol/L	4.10 (3.70–4.60)	4.20 (3.70–4.60)	0.479
Sodium, mmol/L	140.00 (137.00–142.00)	140.00 (137.00–142.00)	0.393
Metabolic markers			
Glucose, mg/dL	133.00 (108.00–167.50)	157.00 (123.00–205.00)	<0.001
Lactate, mmol/L	2.29 (1.50–3.00)	2.50 (1.60–4.10)	<0.001
Blood gas analysis			
PaCO₂, mmHg	41.00 (37.00–46.00)	39.00 (33.00–47.00)	0.014
PaO₂, mmHg	125.00 (85.00–189.00)	114.00 (63.00–219.00)	0.587
Liver function			
Alanine aminotransferase, U/L	43.40 (23.00–85.10)	54.00 (25.00–117.00)	0.022
Aspartate aminotransferase, U/L	67.35 (34.40–131.75)	89.80 (43.00–200.00)	<0.001
Total bilirubin, mg/dL	0.74 (0.50–1.00)	0.78 (0.50–1.14)	0.380
Renal function			
Creatinine, mg/dL	1.10 (0.80–1.60)	1.20 (0.90–1.70)	0.042
Blood urea nitrogen, mg/dL	21.00 (15.00–35.00)	23.00 (15.00–37.00)	0.303
Cardiac injury marker			
Cardiac troponin T, ng/mL	0.05 (0.02–0.14)	0.10 (0.04–0.25)	<0.001
Comorbidities, *n* (%)			
Hypertension	257 (41.45%)	77 (35.48%)	0.122
Acute kidney injury	187 (30.16%)	78 (35.94%)	0.115
Cerebrovascular disease	52 (8.39%)	22 (10.14%)	0.434
Type 2 diabetes	168 (27.10%)	62 (28.57%)	0.675
Hyperlipidemia	172 (27.74%)	45 (20.74%)	0.043
Heart failure	183 (29.52%)	67 (30.88%)	0.706
Therapeutic interventions			
Vasopressor use, *n* (%)	237 (38.23%)	130 (59.91%)	<0.001
Length of stay, days			
Total hospital stay	11.11 (5.96–20.72)	5.64 (2.33–10.04)	<0.001
ICU length of stay	2.99 (1.49–7.37)	3.16 (1.71–6.72)	0.595
Hospital stay within 28 days	11.11 (5.96–20.72)	5.64 (2.33–10.04)	<0.001
In-hospital outcome			
In-hospital mortality (died), *n* (%)	32 (5.16%)	217 (100.00%)	<0.001

Continuous variables are presented as median (interquartile range) and were compared using the Mann–Whitney *U* test; categorical variables are presented as *n* (%) and were compared using the chi-square test.

For categorical variables, only the clinically informative category is listed (female sex; presence of each comorbidity; vasopressor use; in-hospital death). Complementary counts can be derived from the group totals.

In-hospital mortality was 29.75% (249/837); among 28-day survivors, 5.2% died in hospital.

PaCO₂, partial pressure of carbon dioxide; PaO₂, partial pressure of oxygen; ICU, intensive care unit.

In terms of demographic characteristics, the median age of non-survivors was significantly higher than that of survivors (78 vs. 75 years, *p* = 0.048), consistent with the well-established role of advanced age as a key prognostic factor in TBI.

Among vital signs, heart rate was significantly higher in non-survivors than in survivors (93 vs. 90.5 beats/min, *p* = 0.009), while no statistically significant differences were observed in systolic, diastolic, or mean arterial pressure between the two groups. This suggests a more pronounced systemic stress response in patients who died.

Laboratory findings revealed more substantial abnormalities across multiple domains in non-survivors. Inflammatory markers showed significantly elevated white blood cell counts in non-survivors (12.5 × 10^9^/L vs. 9.75 × 10^9^/L, *p* < 0.001). Metabolic parameters were also markedly altered, with higher glucose levels (157 vs. 133 mg/dL, *p* < 0.001) and lactate concentrations (2.5 vs. 2.29 mmol/L, *p* < 0.001). Blood gas analysis showed significantly lower partial pressure of carbon dioxide (PaCO₂) in non-survivors (39.0 vs. 41.0 mmHg, *p* = 0.014), while partial pressure of oxygen (PaO₂) did not differ significantly between groups (*p* = 0.587). Liver function was impaired, as evidenced by significantly higher aspartate aminotransferase (AST; 89.8 vs. 67.35 U/L, *p* < 0.001) and alanine aminotransferase (ALT; 54 vs. 43.4 U/L, *p* = 0.022) levels in non-survivors. Renal function was similarly affected, with significantly elevated creatinine levels (1.2 vs. 1.1 mg/dL, *p* = 0.042).

Cardiac troponin T (cTnT), a highly specific biomarker of myocardial injury, was significantly higher in non-survivors than in survivors (0.10 vs. 0.05 ng/mL, *p* < 0.001).

Regarding therapeutic interventions, vasopressor use was significantly more frequent in non-survivors (59.91% vs. 38.23%, *p* < 0.001), suggesting a greater need for circulatory support. Notably, the prevalence of hyperlipidemia was significantly lower in non-survivors (20.74% vs. 27.74%, *p* = 0.043).

Analysis of hospitalization duration showed that both total length of stay and length of stay within the first 28 days were significantly shorter in non-survivors (*p* < 0.001), which is likely attributable to early mortality.

Patients were identified from the MIMIC-IV and MIMIC-III databases to form the development cohort, and from Taihe Hospital to form the external validation cohort. Patients were excluded if they had a diagnosis of myocardial infarction or lacked cardiac troponin T (cTnT) measurements on the first ICU day. The final development cohort included 837 patients (217 non-survivors and 620 survivors within 28 days). The external validation cohort ultimately included 121 patients after excluding those with undetermined survival status or pre-admission myocardial infarction.

### Restricted cubic spline analysis

The unadjusted model (Figure 2) revealed a significant nonlinear relationship between cTnT and 28-day mortality (P for nonlinearity < 0.001). After adjusting for age and Glasgow Coma Scale (GCS) score (Figure 2B), this nonlinear pattern remained statistically significant (P for nonlinearity < 0.001), indicating that the prognostic effect of cTnT is independent of these two key confounders.

**Figure 2:**
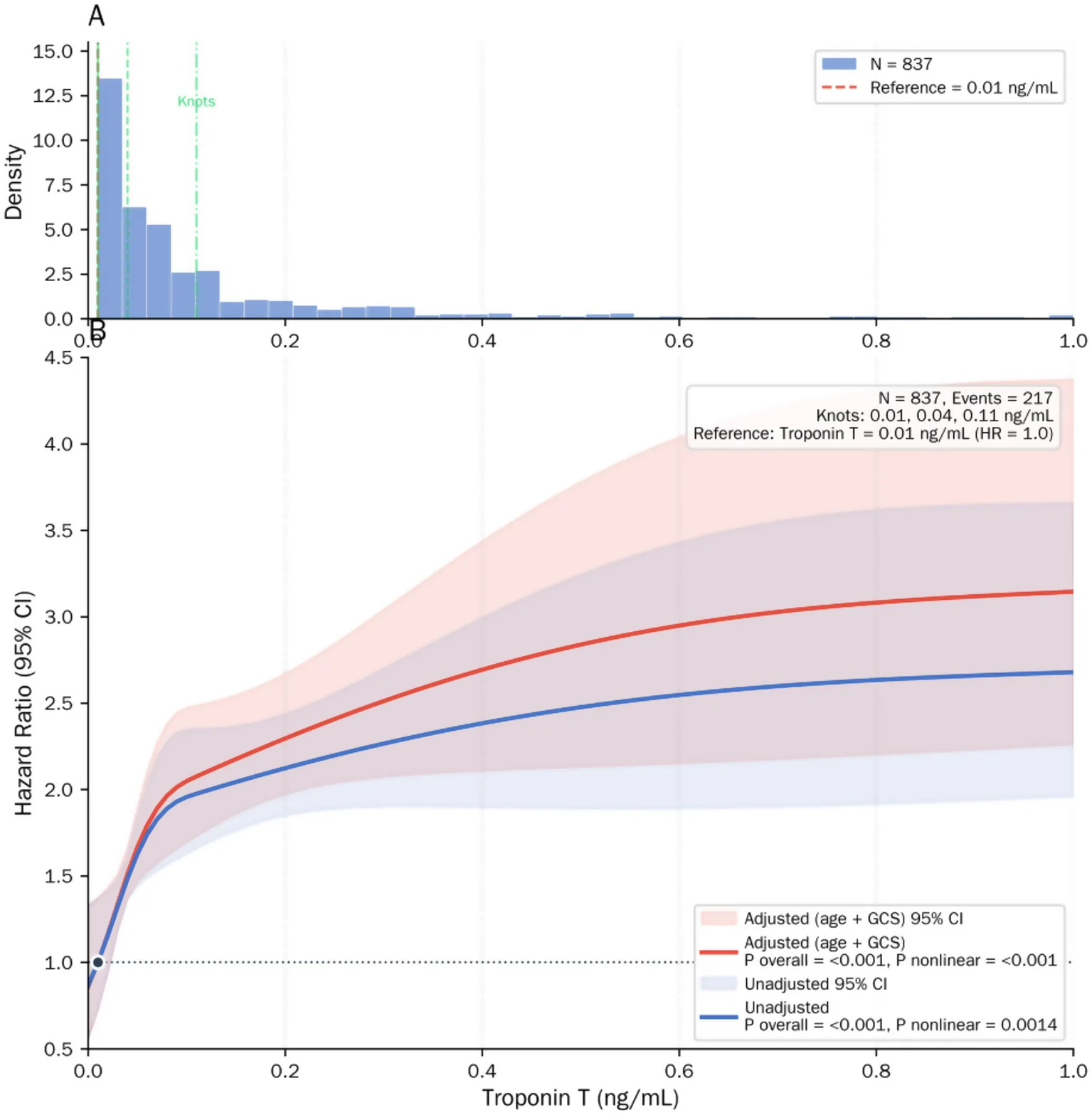
Restricted cubic spline analysis of the association between cTnT and 28-day mortality. **(A)** Density distribution of cTnT values (highly right-skewed, *n* = 837). The dashed green line indicates the knot location (0.11 ng/mL), and the dashed red line represents the reference value (0.01 ng/mL). **(B)** RCS curves illustrating the dose–response association between cTnT and 28-day mortality. Knots were placed at 0.01, 0.04, and 0.11 ng/mL. Troponin T = 0.01 ng/mL was set as the reference (HR = 1.0). The blue line represents the unadjusted model, and the red line represents the model adjusted for age and Glasgow Coma Scale (GCS) score. Shaded areas represent the corresponding 95% confidence intervals (95% CI). P overall < 0.001 for both models.

As shown in Figure 2B, the hazard ratio (HR) for mortality increased progressively with rising cTnT. In the low-concentration range (<0.1 ng/mL), the HR rose sharply; beyond approximately 0.1 ng/mL, the curve flattened slightly but continued to increase, suggesting that mortality risk remains elevated at higher cTnT levels. The distribution of cTnT values was right-skewed, with the majority of patients having levels below 0.10 ng/mL.

These results indicate that the association between cTnT and 28-day mortality in TBI patients is robustly nonlinear.

### Baseline characteristics by cTnT quartile groups

Analysis of baseline characteristics according to cardiac troponin T (cTnT) quartile groups revealed significant differences across multiple clinical parameters.

Regarding demographic features, median age decreased progressively with increasing cTnT levels, from 78 years in Q1 to 68 years in Q4 (*p* < 0.001).

Vital sign parameters demonstrated that the highest cTnT quartile (Q4) had significantly elevated heart rate (93.95 beats/min, *p* = 0.017) and lower systolic blood pressure (125.20 mmHg, *p* = 0.018) compared with other quartiles, suggesting more pronounced hemodynamic instability in this cohort.

Laboratory parameters displayed significant trends across cTnT quartiles.

Inflammatory markers showed progressive elevation in white blood cell count from Q1 (9.80 × 10^9^/L) to Q4 (11.60 × 10^9^/L; *p* < 0.001).

Metabolic parameters revealed increasing blood glucose levels from Q1 (135 mg/dL) to Q4 (149 mg/dL; *p* < 0.001).

Liver function tests showed a progressive increase in aspartate aminotransferase (AST) levels from Q1 (68 U/L) to Q4 (90.05 U/L; *p* = 0.016), while total bilirubin levels decreased progressively (*p* = 0.007).

Renal function assessment showed progressively higher creatinine levels from Q1 (1.00 mg/dL) to Q4 (1.20 mg/dL; *p* < 0.001).

Therapeutic interventions reflected clinical severity, with vasopressor utilization rates increasing progressively across quartiles from Q1 (33.96%) to Q4 (60.68%; *p* < 0.001), indicating greater circulatory support requirements among patients with higher cTnT levels.

Comorbidity distribution showed significant interquartile differences.

Hypertension prevalence declined with increasing cTnT levels (Q1–Q4: 51.32, 38.36, 35.27, and 31.07%, respectively; *p* < 0.001).

Acute kidney injury incidence increased progressively from Q1 (24.15%) to Q4 (38.35%; *p* = 0.009).

Clinical outcomes exhibited a clear mortality gradient corresponding to cTnT levels.

In-hospital mortality increased from Q1 (20.38%) to Q4 (37.86%; *p* < 0.001), and 28-day mortality rose similarly from Q1 (17.74%) to Q4 (33.98%; *p* < 0.001).

Furthermore, both hospital length of stay and ICU length of stay were significantly shorter in the Q4 group compared with other quartiles (*p* = 0.012 and *p* = 0.004, respectively). Baseline characteristics stratified by cTnT quartile groups are summarized in Table 2.

**Table 2:** Baseline characteristics of traumatic brain injury patients stratified by cardiac troponin T (cTnT) quartile.

Variable	Q1 (*n* = 265)	Q2 (*n* = 159)	Q3 (*n* = 207)	Q4 (*n* = 206)	*p*-value
Demographics					
Age, years	78.00 (61.00–84.00)	77.00 (63.00–87.00)	75.00 (62.00–85.00)	68.00 (56.00–80.00)	<0.001
Female sex, *n* (%)	94 (35.47%)	64 (40.25%)	76 (36.71%)	69 (33.50%)	0.600
Vital signs					
Heart rate, beats/min	90.50 (77.00–100.95)	90.00 (78.00–101.00)	90.00 (78.00–101.00)	93.95 (82.00–105.20)	0.017
Systolic blood pressure, mmHg	133.30 (116.00–146.00)	133.00 (116.00–146.00)	132.00 (115.00–145.00)	125.20 (112.00–144.00)	0.018
Diastolic blood pressure, mmHg	70.00 (61.40–80.00)	72.00 (62.00–80.00)	71.50 (61.00–80.00)	72.00 (61.00–78.60)	0.243
Mean arterial pressure, mmHg	85.40 (77.00–97.00)	88.00 (78.00–95.00)	87.00 (77.00–95.60)	84.00 (76.00–94.00)	0.115
Respiratory rate, breaths/min	18.10 (16.00–22.00)	19.30 (16.00–22.00)	20.00 (16.00–22.00)	19.95 (16.00–22.00)	0.044
Neurological status					
Glasgow Coma Scale score	14.00 (13.00–15.00)	14.00 (13.00–15.00)	1.00 (13.00–15.00)	15.00 (13.00–15.00)	0.095
Hematology					
Hemoglobin, g/dL	11.00 (9.30–12.20)	10.30 (9.30–12.30)	10.50 (9.30–12.30)	10.70 (9.40–12.60)	0.270
Platelet count, ×10^9^/L	182.00 (140.00–236.00)	178.90 (138.00–236.00)	186.00 (140.00–236.00)	182.50 (129.00–239.00)	0.597
White blood cell count, ×10^9^/L	9.80 (7.30–13.45)	9.80 (7.30–13.45)	10.80 (7.60–14.10)	11.60 (9.40–17.20)	<0.001
Electrolytes					
Total calcium, mg/dL	8.40 (7.90–9.00)	8.40 (7.80–9.00)	8.50 (7.90–9.00)	8.50 (7.60–9.00)	0.893
Potassium, mmol/L	4.00 (3.70–4.60)	4.00 (3.70–4.60)	4.40 (3.70–4.60)	4.10 (3.70–4.60)	<0.001
Sodium, mmol/L	139.00 (137.00–142.00)	140.00 (137.00–142.00)	140.00 (137.00–142.00)	140.00 (137.00–142.00)	0.131
Metabolic markers					
Glucose, mg/dL	135.00 (108.00–167.50)	128.00 (108.00–167.50)	145.00 (111.00–175.00)	149.00 (123.00–205.00)	<0.001
Lactate, mmol/L	2.30 (1.50–3.00)	2.20 (1.50–3.00)	2.30 (1.50–3.20)	2.30 (1.60–4.10)	0.186
Blood gas analysis					
PaCO₂, mmHg	40.90 (37.00–46.00)	40.10 (36.00–46.00)	40.90 (37.00–46.00)	40.00 (33.00–47.00)	0.841
PaO₂, mmHg	137.30 (85.00–189.00)	129.60 (85.00–189.00)	114.00 (79.00–195.00)	112.00 (63.00–219.00)	<0.001
Liver function					
Alanine aminotransferase, U/L	41.50 (23.00–85.10)	41.00 (23.00–85.10)	49.00 (25.00–117.00)	55.00 (25.00–117.00)	0.067
Aspartate aminotransferase, U/L	68.00 (34.40–131.75)	56.00 (34.40–131.75)	73.00 (37.00–141.00)	90.05 (43.00–200.00)	0.016
Total bilirubin, mg/dL	0.80 (0.50–1.00)	0.70 (0.50–1.00)	0.70 (0.50–1.03)	0.70 (0.50–1.14)	0.007
Renal function					
Creatinine, mg/dL	1.00 (0.80–1.60)	1.00 (0.80–1.60)	1.20 (0.90–1.70)	1.20 (0.90–1.70)	<0.001
Blood urea nitrogen, mg/dL	20.00 (15.00–35.00)	22.00 (15.00–35.00)	27.00 (15.00–37.00)	21.00 (15.00–37.00)	<0.001
Cardiac injury marker					
Cardiac troponin T, ng/mL	0.02 (0.01–0.03)	0.05 (0.04–0.06)	0.10 (0.07–0.17)	0.40 (0.18–14.20)	<0.001
Comorbidities, *n* (%)					
Hypertension	136 (51.32%)	61 (38.36%)	73 (35.27%)	64 (31.07%)	<0.001
Acute kidney injury	64 (24.15%)	53 (33.33%)	69 (33.33%)	79 (38.35%)	0.009
Cerebrovascular disease	20 (7.55%)	15 (9.43%)	20 (9.66%)	19 (9.22%)	0.842
Type 2 diabetes	79 (29.81%)	41 (25.79%)	61 (29.47%)	49 (23.79%)	0.428
Hyperlipidemia	86 (32.45%)	32 (20.13%)	45 (21.74%)	54 (26.21%)	0.014
Heart failure	67 (25.28%)	48 (30.19%)	67 (32.37%)	68 (33.01%)	0.235
Therapeutic interventions					
Vasopressor use, *n* (%)	90 (33.96%)	60 (37.74%)	92 (44.44%)	125 (60.68%)	<0.001
Length of stay, days					
Total hospital stay	9.73 (5.96–20.72)	10.68 (5.96–20.72)	8.90 (4.93–17.24)	9.25 (2.33–10.04)	0.382
ICU length of stay	2.95 (1.49–7.37)	2.58 (1.49–7.37)	3.12 (1.51–7.00)	3.81 (1.71–6.72)	0.018
Hospital stay within 28 days	9.73 (5.96–20.72)	10.68 (5.96–20.72)	8.90 (4.93–17.24)	9.25 (2.33–10.04)	0.387
Outcomes					
In-hospital mortality (died), *n* (%)	54 (20.38%)	40 (25.16%)	77 (37.20%)	78 (37.86%)	<0.001
28-day mortality (died), *n* (%)	47 (17.74%)	29 (18.24%)	71 (34.30%)	70 (33.98%)	<0.001
Hospital survival time †, days	7.78 (5.96–20.72)	12.25 (5.96–20.72)	6.76 (4.93–17.24)	3.90 (2.33–10.04)	0.012
ICU survival time †, days	7.76 (5.96–20.72)	12.22 (5.96–20.72)	6.67 (4.93–17.24)	3.80 (2.33–10.04)	0.004

Continuous variables are presented as median (interquartile range) on two lines and were compared using the Kruskal–Wallis test; categorical variables are presented as n (%) and were compared using the chi-square test. A single global P value is reported for each variable, indicating whether any significant difference exists across the four quartile groups.

Quartile cut-offs: Q1 ≤ 0.03 ng/mL; Q2 0.03–0.06 ng/mL; Q3 0.06–0.17 ng/mL; Q4 > 0.17 ng/mL.

^†^Survival time was calculated only for patients who died (time from admission to death); for survivors, survival time was censored at 28 days.

For categorical variables, only the clinically informative category is listed; complementary counts can be derived from the group totals.

PaCO₂, partial pressure of carbon dioxide; PaO₂, partial pressure of oxygen; ICU, intensive care unit.

### Boruta feature selection

Before applying LASSO regression for final variable selection, we performed a Boruta analysis to identify all features that exhibit a statistically significant association with 28-day mortality. The Boruta algorithm generates “shadow” variables by randomly permuting the original predictors and compares the importance of each real variable against the maximum importance among shadow variables across multiple iterations. Variables that consistently outperform the best shadow variable are classified as Confirmed, those that perform significantly worse are Rejected, and borderline cases are labeled Tentative.

Among the 31 candidate variables entered into Boruta, 22 were confirmed as relevant predictors of 28-day mortality (Figure 3). The confirmed variables included demographic characteristics (age), neurological status (Glasgow Coma Scale), markers of systemic inflammation (white blood cell count), metabolic and hemodynamic parameters (bedside glucose, lactate, heart rate, mean arterial pressure, systolic blood pressure), blood gas parameters (PaO₂, PaCO₂), indicators of liver and renal function (AST, ALT, total bilirubin, creatinine, blood urea nitrogen), electrolytes and hematologic indices (calcium, sodium, hemoglobin, platelet count), as well as cTnT quartile group and vasopressor use. Eight variables—potassium, gender, hyperlipidemia, acute kidney injury, heart failure, cerebrovascular disease, type 2 diabetes, and hypertension—were rejected by Boruta and excluded from subsequent analyses. One variable (diastolic blood pressure, norm Hits = 0.58) was classified as Tentative after the maximum number of iterations.

**Figure 3:**
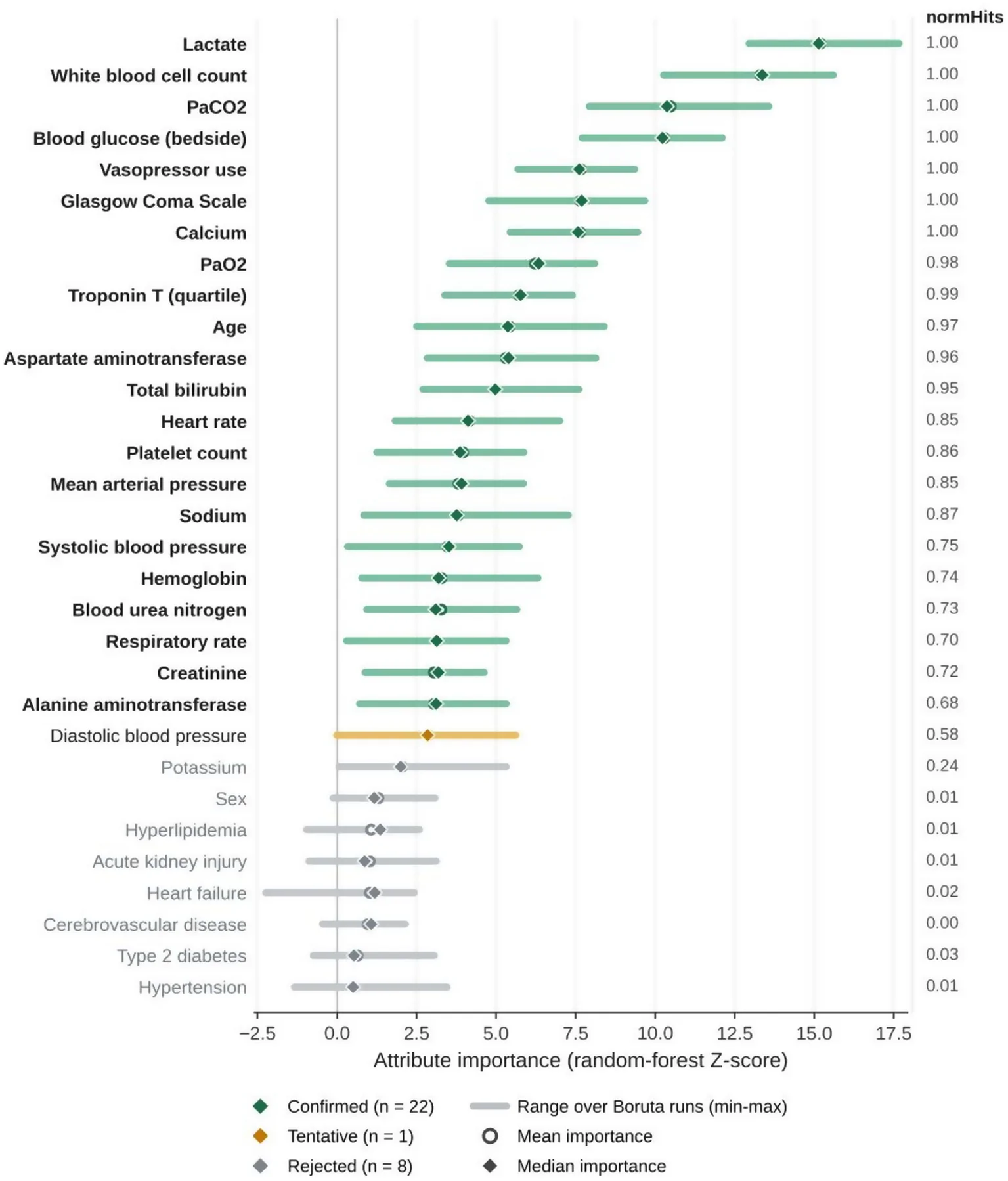
Boruta variable importance plot.

The horizontal bar plot in Figure 3 displays the random-forest Z-scores for each variable, with Confirmed variables shown as green diamonds (*n* = 22), the Tentative variable as orange diamonds (*n* = 1), and Rejected variables as gray diamonds (*n* = 8). Horizontal lines indicate the range of importance scores across Boruta runs, and the rightmost column shows the normHits proportion for each variable. This two-stage filtering approach ensures that only robust and biologically plausible predictors are passed to the LASSO regression for further shrinkage and selection. The detailed Boruta variable importance scores are provided in Supplementary Table S4. The coefficients of the 13 selected variables at λ.min are listed in Supplementary Table S7.

Horizontal bar plot of random-forest Z-scores for each variable. Confirmed predictors (*n* = 22) are shown in green, the Tentative predictor (*n* = 1) in orange, and Rejected variables (*n* = 8) in gray. Horizontal lines represent the range of importance scores across Boruta runs. The rightmost column shows the normHits proportion for each variable.

### LASSO regression

The 22 predictors confirmed (plus 1 tentative) by Boruta were entered into a LASSO regression with 10-fold cross-validation. The coefficient paths of all variables as a function of the penalty parameter λ are shown in Figure 4A. As λ increases, coefficients of less informative predictors shrink progressively toward zero, while the most robust predictors retain non-zero coefficients for longer.

**Figure 4:**
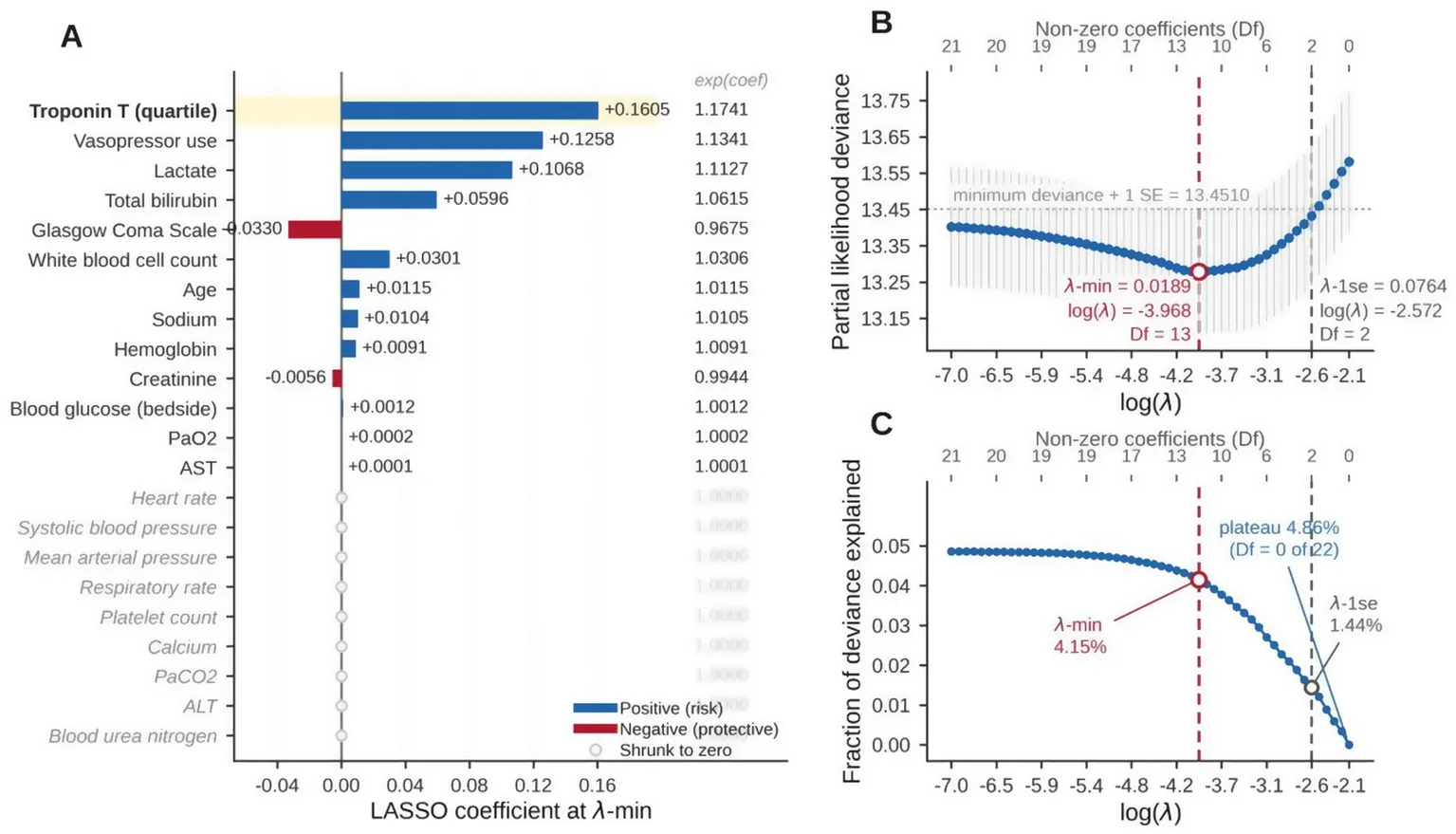
LASSO regression analysis. **(A)** Coefficient paths of all variables as a function of the penalty parameter λ, with blue bars representing positive coefficients (risk factors) and red bars representing negative coefficients (protective factors). Gray circles indicate variables whose coefficients were shrunk to zero. The rightmost column shows exp.(coef) values. **(B)** Cross-validation curve showing partial likelihood deviance across a range of λ values. The optimal λ (λ.min, red dashed line) was selected at LogLambda = −3.875, corresponding to 13 non-zero coefficients. The λ.1se (black dashed line) represents the most parsimonious model within one standard error of the minimum. **(C)** Fraction of deviance explained by the model across λ values, with λ.min corresponding to 4.15% deviance explained.

The cross-validation curve (Figure 4B) identified the optimal λ at λ.min = 0.02076 (LogLambda = −3.875), which minimized the cross-validated partial likelihood deviance. At this penalty level, 13 variables retained non-zero coefficients and were selected for the final model: age, Glasgow Coma Scale (GCS), hemoglobin, white blood cell count, bedside glucose, sodium, lactate, partial pressure of oxygen (PaO₂), aspartate aminotransferase (AST), total bilirubin, creatinine, cTnT quartile group, and vasopressor use. The coefficients of the 13 selected variables at λ.min are listed in Supplementary Table S7.

### Association between cTnT and 28-day all-cause mortality

In multivariable Cox regression, after adjusting for the 12 covariates selected by LASSO (excluding cTnT quartile itself), cTnT quartile remained independently associated with 28-day mortality. Using Q1 (≤0.03 ng/mL) as reference, patients in Q3 (0.06–0.17 ng/mL) and Q4 (>0.17 ng/mL) had significantly elevated risks of death (HR = 2.01, 95% CI: 1.37–2.95, *p* < 0.001; and HR = 1.96, 95% CI: 1.31–2.93, *p* = 0.001, respectively), while Q2 (0.03–0.06 ng/mL) did not differ from Q1 (HR = 1.12, 95% CI: 0.70–1.80, *p* = 0.626).

Among other covariates, age (per 1-year increase: HR = 1.021, 95% CI: 1.012–1.030, *p* < 0.001), white blood cell count (per 1 × 10₉/L increase: HR = 1.033, 95% CI: 1.017–1.048, *p* < 0.001), lactate (per 1 mmol/L increase: HR = 1.114, 95% CI: 1.054–1.178, *p* < 0.001), total bilirubin (per 1 mg/dL increase: HR = 1.108, 95% CI: 1.048–1.172, *p* < 0.001), bedside glucose (per 1 mg/dL increase: HR = 1.002, 95% CI: 1.001–1.003, *p* = 0.001), sodium (per 1 mmol/L increase: HR = 1.028, 95% CI: 1.003–1.054, *p* = 0.025), and vasopressor use (HR = 1.394, 95% CI: 1.032–1.881, *p* = 0.030) were independently associated with increased mortality. In contrast, higher GCS score was protective (per 1-point increase: HR = 0.945, 95% CI: 0.909–0.983, *p* = 0.005). Hemoglobin, PaO₂, AST, and creatinine did not reach statistical significance in the fully adjusted model (see Table 3).

**Table 3:** Multivariable Cox regression analysis of cTnT quartiles and 28-day all-cause mortality.

cTnT quartile group	Adjusted HR (95% CI)	*p*-value
Q1 (≤0.03 ng/mL)	Reference	—
Q2 (0.03–0.06 ng/mL)	1.124 (0.703–1.797)	0.626
Q3 (0.06–0.17 ng/mL)	2.009 (1.369–2.948)	<0.001
Q4 (>0.17 ng/mL)	1.956 (1.307–2.927)	0.001

Adjusted for the 12 covariates retained by LASSO after excluding the cTnT quartile group itself: age, Glasgow Coma Scale (GCS) score, hemoglobin, white blood cell count, bedside glucose, sodium, lactate, partial pressure of oxygen (PaO₂), aspartate aminotransferase (AST), total bilirubin, creatinine, and vasopressor use. HR, hazard ratio; CI, confidence interval.

Univariate results are provided in Supplementary Table S2; the full multivariable model for all covariates is provided in Supplementary Table S5.

### Subgroup analyses

To further evaluate the robustness of the association between cTnT quartiles and 28-day mortality across diverse clinical populations, we performed prespecified subgroup analyses stratified by eight clinically relevant factors: gender, vasopressor (VP) use, hypertension (HTN), acute kidney injury (AKI), cerebrovascular accident (CVA), type 2 diabetes mellitus (T2DM), hyperlipidemia (HLD), and heart failure (HF). These subgroups were selected *a priori* based on their established relevance to TBI prognosis and cTnT metabolism.

As shown in Figure 5, the association between higher cTnT quartiles and increased 28-day mortality was consistent across all prespecified subgroups, with all *p* values for interaction exceeding 0.05 (range: 0.092–0.938). Specifically, patients in Q3 demonstrated significantly elevated hazard ratios compared with Q1 in 11 of 16 subgroup strata, and patients in Q4 showed significantly elevated hazard ratios in 10 of 16 strata. A graded dose–response relationship was evident, with Q4 consistently demonstrating point estimates equal to or exceeding those of Q3 in the majority of strata.

**Figure 5:**
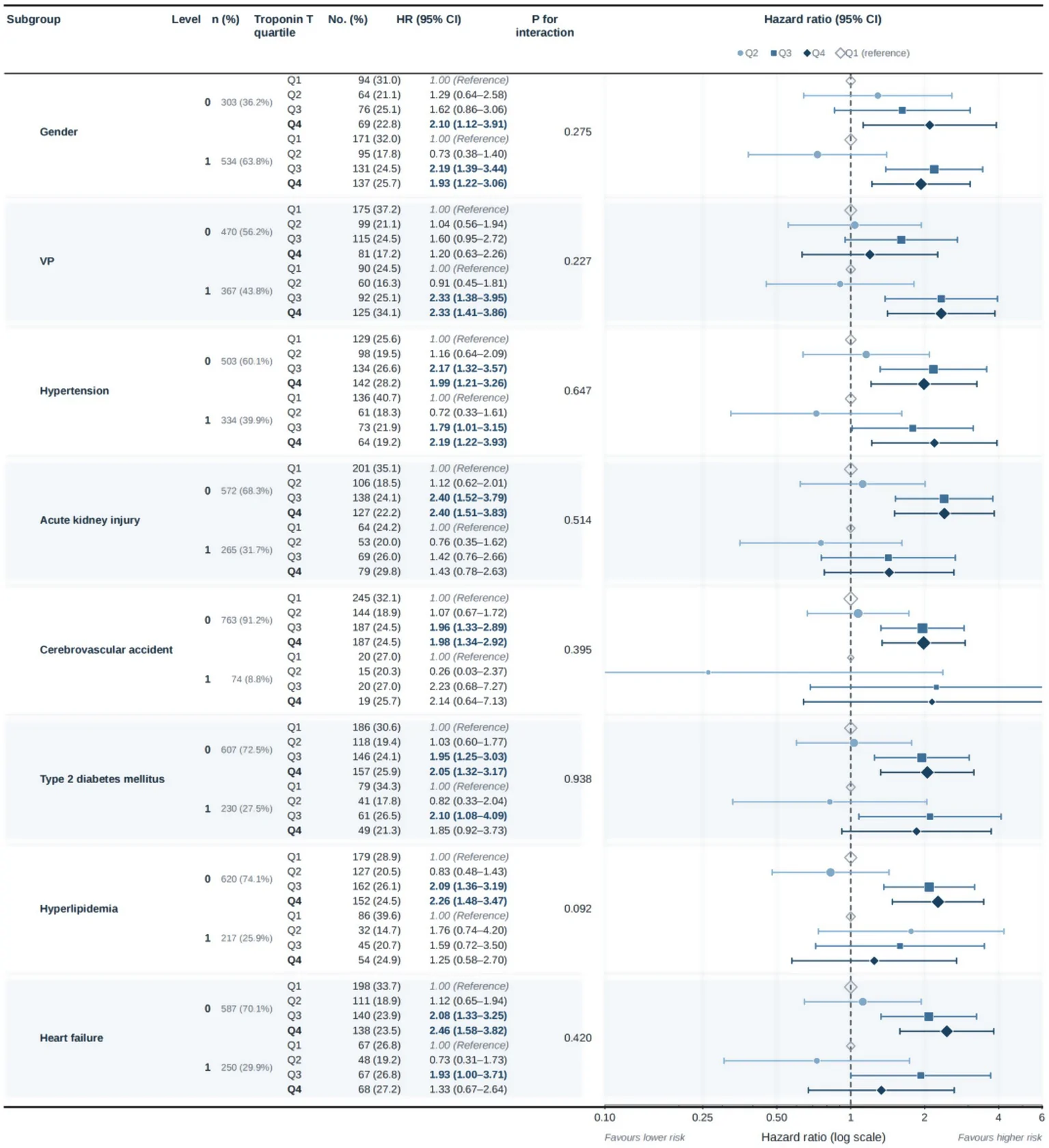
Subgroup analysis of the association between cardiac troponin T quartiles and 28-day all-cause mortality.

Notably, in patients with heart failure (29.9% of the cohort), the direction of the association was consistent with the overall findings, although wider confidence intervals reflected the smaller subgroup sample size. Similarly, in patients with acute kidney injury (31.7% of the cohort), both Q3 (HR = 2.33, 95% CI: 1.38–3.95) and Q4 (HR = 2.33, 95% CI: 1.41–3.86) were significantly associated with increased mortality compared with Q1. These results demonstrate that the prognostic value of cTnT is preserved across patients with varying comorbidity burdens, supporting the generalizability of our findings to broader TBI populations.

Forest plot showing hazard ratios (HRs) with 95% confidence intervals (CIs) for 28-day mortality across prespecified subgroups. Cox proportional-hazards regression was used, with troponin T quartile 1 (Q1) as the reference within each subgroup stratum. Quartiles were derived from the entire analysis set (*n* = 837): Q1 = 265 (31.7%), Q2 = 159 (19.0%), Q3 = 207 (24.7%), Q4 = 206 (24.6%). The marker area is proportional to the number of patients in the stratum, and marker color and shape encode the quartile. The horizontal axis is logarithmic, and the dashed vertical line marks HR = 1.00. All *p* values for interaction exceeded 0.05 (range: 0.092–0.938), indicating no statistically significant effect modification by any of the prespecified subgroups. The consistent direction of association across subgroups supports the robustness of the prognostic value of cTnT in diverse TBI populations. Abbreviations: AKI, acute kidney injury; CI, confidence interval; CVA, cerebrovascular accident; HF, heart failure; HLD, hyperlipidemia; HR, hazard ratio; HTN, hypertension; Q, quartile; T2DM, type 2 diabetes mellitus; VP, vasopressor.

### Association between cTnT and in-hospital all-cause mortality

To assess the robustness of the primary findings, we further examined the association between cTnT levels and in-hospital all-cause mortality. In multivariable Cox regression adjusting for the 12 LASSO-selected covariates (excluding cTnT quartile), cTnT quartile remained independently associated with in-hospital death. Using Q1 (≤0.03 ng/mL) as reference, patients in Q3 (0.06–0.17 ng/mL) had a nearly threefold increased risk of in-hospital mortality (HR = 2.92, 95% CI: 1.42–5.99, *p* = 0.003), and those in Q4 (>0.17 ng/mL) also had a significantly elevated risk (HR = 2.50, 95% CI: 1.08–5.75, *p* = 0.032). The Q2 group (0.03–0.06 ng/mL) showed a trend toward higher risk but did not reach statistical significance (HR = 2.04, 95% CI: 0.89–4.67, *p* = 0.090). Univariate results are presented in Supplementary Table S2.

Among other covariates, age (per 1-year increase: HR = 1.058, 95% CI: 1.033–1.083, *p* < 0.001), white blood cell count (per 1 × 10₉/L increase: HR = 1.050, 95% CI: 1.025–1.075, *p* < 0.001), bedside glucose (per 1 mg/dL increase: HR = 1.003, 95% CI: 1.001–1.005, *p* = 0.001), sodium (per 1 mmol/L increase: HR = 1.046, 95% CI: 1.010–1.083, *p* = 0.012), and AST (per 1 U/L increase: HR = 1.001, 95% CI: 1.000–1.001, *p* = 0.011) were independently associated with increased in-hospital mortality. In contrast, higher GCS score was protective (HR = 0.883, 95% CI: 0.819–0.953, *p* = 0.001). Vasopressor use showed an inverse association with in-hospital death (HR = 0.507, 95% CI: 0.281–0.912, *p* = 0.023). Hemoglobin, PaO₂, creatinine, total bilirubin, and lactate did not reach statistical significance in the fully adjusted model (see Table 4).

**Table 4:** Multivariable Cox regression analysis of cTnT quartiles and in-hospital all-cause mortality.

cTnT quartile group	Adjusted HR (95% CI)	*p*-value
Q1 (≤0.03 ng/mL)	Reference	—
Q2 (0.03–0.06 ng/mL)	2.043 (0.895–4.666)	0.090
Q3 (0.06–0.17 ng/mL)	2.921 (1.424–5.992)	0.003
Q4 (>0.17 ng/mL)	2.495 (1.084–5.747)	0.032

Adjusted for the same 12 LASSO-retained covariates as Table 3. HR, hazard ratio; CI, confidence interval. Note: Vasopressor-use inverse association is interpreted with caution due to potential immortal-time bias, see Discussion section.

Univariate results are provided in Supplementary Table S2; the full multivariable model for in-hospital mortality is provided in Supplementary Table S6.

### Kaplan–Meier survival analysis

Patients were stratified into four groups based on cTnT quartiles (as defined in Table 2): Q1 (≤0.03 ng/mL), Q2 (0.03–0.06 ng/mL), Q3 (0.06–0.17 ng/mL), and Q4 (>0.17 ng/mL). Kaplan–Meier analysis revealed significant heterogeneity in 28-day survival across the groups (overall Log-rank *p* < 0.001).

Pairwise comparisons using the Log-rank test showed that patients in Q2 had similar survival probabilities to those in Q1 (*p* = 0.896). In contrast, both Q3 and Q4 had significantly worse outcomes compared with Q1 (*p* = 0.0001 and *p* = 0.0002, respectively). Q3 also differed significantly from Q2 (*p* = 0.0008), and Q4 differed from Q2 (*p* = 0.0010). However, no statistically significant difference was observed between Q3 and Q4 (*p* = 0.992), suggesting a threshold effect whereby cTnT levels exceeding 0.06 ng/mL are associated with a similarly elevated mortality risk.

Kaplan–Meier survival curves for patients stratified by cardiac troponin T (cTnT) quartiles (*n* = 837). The x-axis represents time after hospital admission (days), and the y-axis represents survival probability. Group definitions: Q1 (≤0.03 ng/mL, *n* = 265), Q2 (0.03–0.06 ng/mL, *n* = 159), Q3 (0.06–0.17 ng/mL, *n* = 207), and Q4 (>0.17 ng/mL, *n* = 206). Shaded areas represent 95% confidence intervals. Tick marks indicate censored observations. The overall log-rank test confirmed significant differences in survival across groups (*p* < 0.001). Pairwise comparisons: Q3 vs. Q1, *p* = 0.0001; Q4 vs. Q1, *p* = 0.0002; Q3 vs. Q2, *p* = 0.0008; Q4 vs. Q2, *p* = 0.0010; Q3 vs. Q4, *p* = 0.992. The number of patients at risk at days 0, 7, 14, 21, and 28 is shown in the table below the Figure 6.

**Figure 6:**
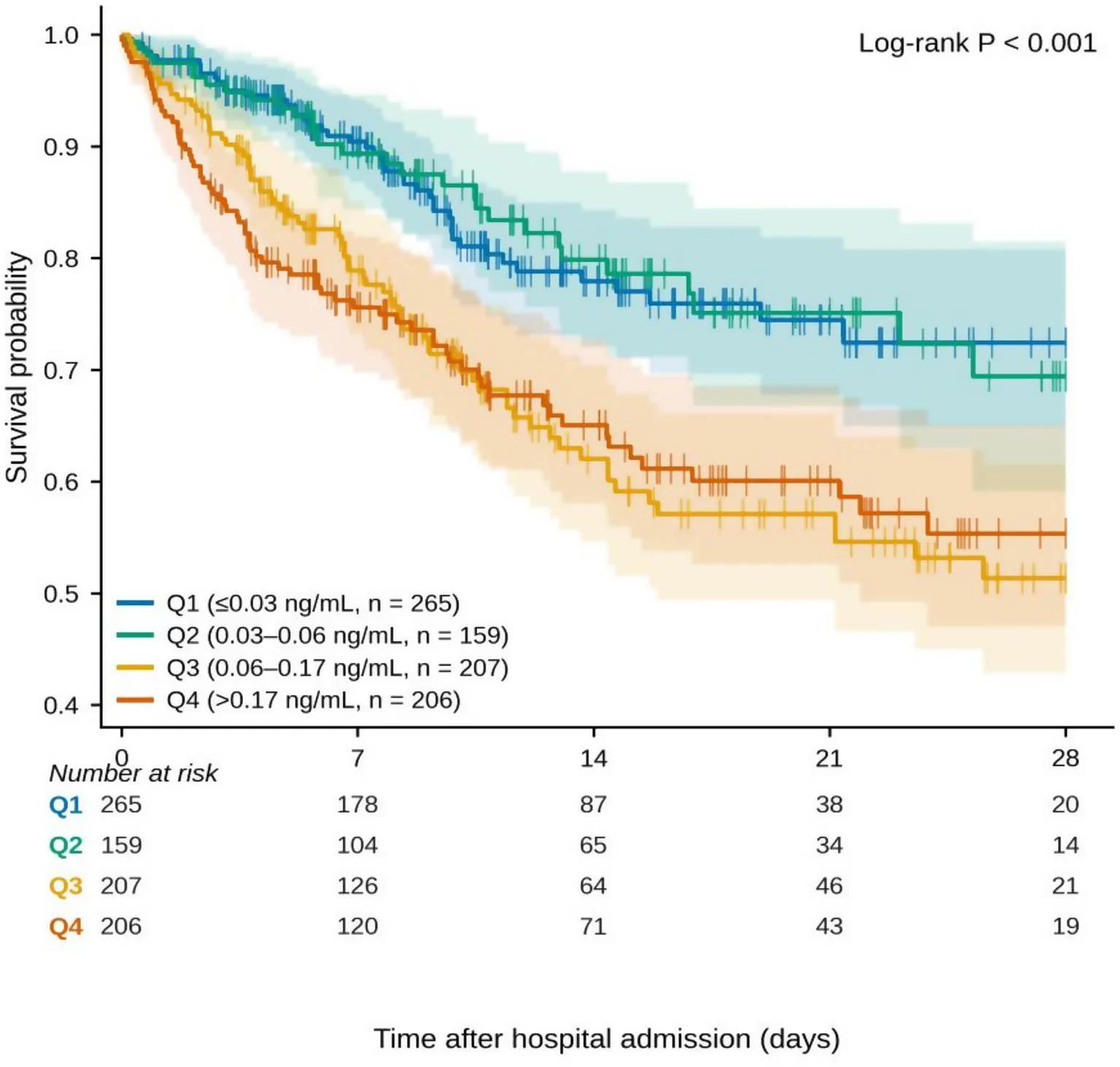
Kaplan–Meier survival curves for patients stratified by cardiac troponin T (cTnT) quartiles (*n* = 837).

### Model performance and selection

To evaluate the predictive performance for 28-day mortality in TBI patients, five machine learning algorithms—Random Forest, Bayesian Additive Regression Trees (BART), Gradient Boosting Machine (GBM), Conditional Inference Tree (CTREE), and Support Vector Machine (SVM)—were trained on the 13 LASSO-selected variables. LASSO identified 13 predictors including cTnT quartile; in Cox regression models, cTnT quartile was the exposure of interest and the remaining 12 variables were included as covariates. Machine learning models used all 13 variables. Their receiver operating characteristic (ROC) curves and corresponding area under the curve (AUC) values are shown in Figure 7. Among all models, Random Forest achieved the highest AUC of 0.783 (95% CI: 0.701–0.865), followed by BART (AUC = 0.753, 95% CI: 0.669–0.838), GBM (AUC = 0.751, 95% CI: 0.669–0.833), SVM (AUC = 0.650, 95% CI: 0.549–0.751), and CTREE (AUC = 0.568, 95% CI: 0.455–0.681). Based on this superior discriminative performance, Random Forest was selected as the optimal predictive model for subsequent analyses. Detailed performance metrics are provided in Supplementary Table S3 (hyperparameters) and Supplementary Table S8 (model performance comparison).

**Figure 7:**
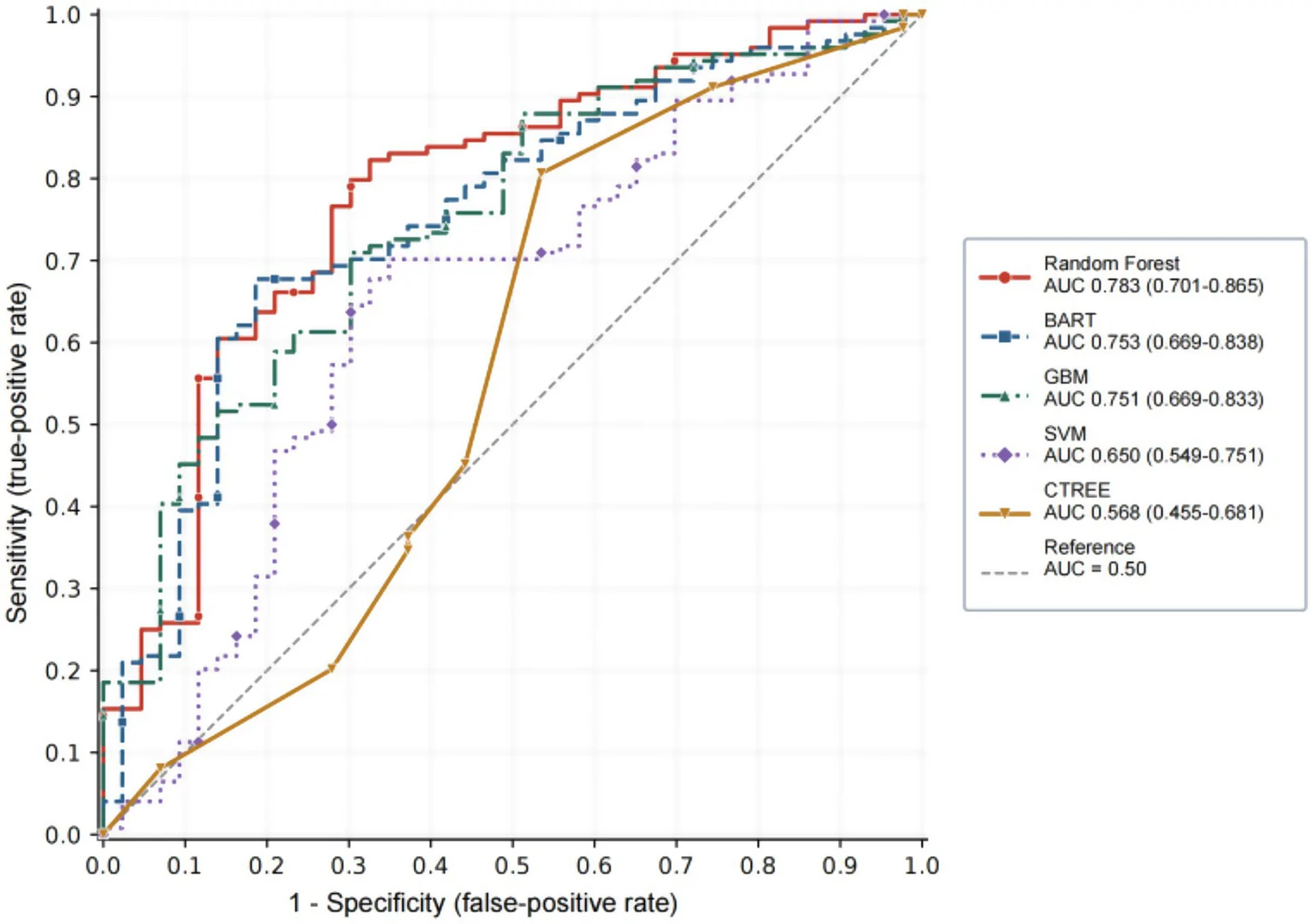
Receiver operating characteristic (ROC) curves of five machine learning models for predicting 28-day all-cause mortality in the internal test set (*n* = 251). The models were trained using the 13 LASSO-selected variables. Area under the curve (AUC) values with 95% confidence intervals: Random Forest, 0.783 (0.701–0.865); BART, 0.753 (0.669–0.838); GBM, 0.751 (0.669–0.833); SVM, 0.650 (0.549–0.751); CTREE, 0.568 (0.455–0.681). The dashed diagonal line represents a random classifier (AUC = 0.50).

### Model calibration and clinical utility

To comprehensively evaluate the predictive performance of the five machine learning models, we assessed calibration, overall accuracy (Brier score), and clinical utility (decision curve analysis). Calibration curves constructed by quintile stratification demonstrated acceptable agreement between predicted and observed 28-day mortality rates across all models (Figure 8). After logistic recalibration, the Brier scores ranged from 0.155 (Random Forest) to 0.186 (CTREE), all notably lower than the null model Brier score of 0.191. The corresponding scaled Brier scores (i.e., 1 − Brier/Brier_null) ranged from 0.028 (CTREE) to 0.192 (Random Forest), indicating that all models provided incremental predictive value beyond the intercept-only model, with Random Forest achieving the best overall calibration-discrimination performance. Detailed model performance metrics including AUC, calibration slope, and Brier scores are provided in Supplementary Table S8. Notably, several alternative algorithms (BART, SVM) showed highly inflated calibration slopes, indicating poor risk calibration despite acceptable discrimination; Random Forest offered the best balance of discrimination and calibration performance.

**Figure 8:**
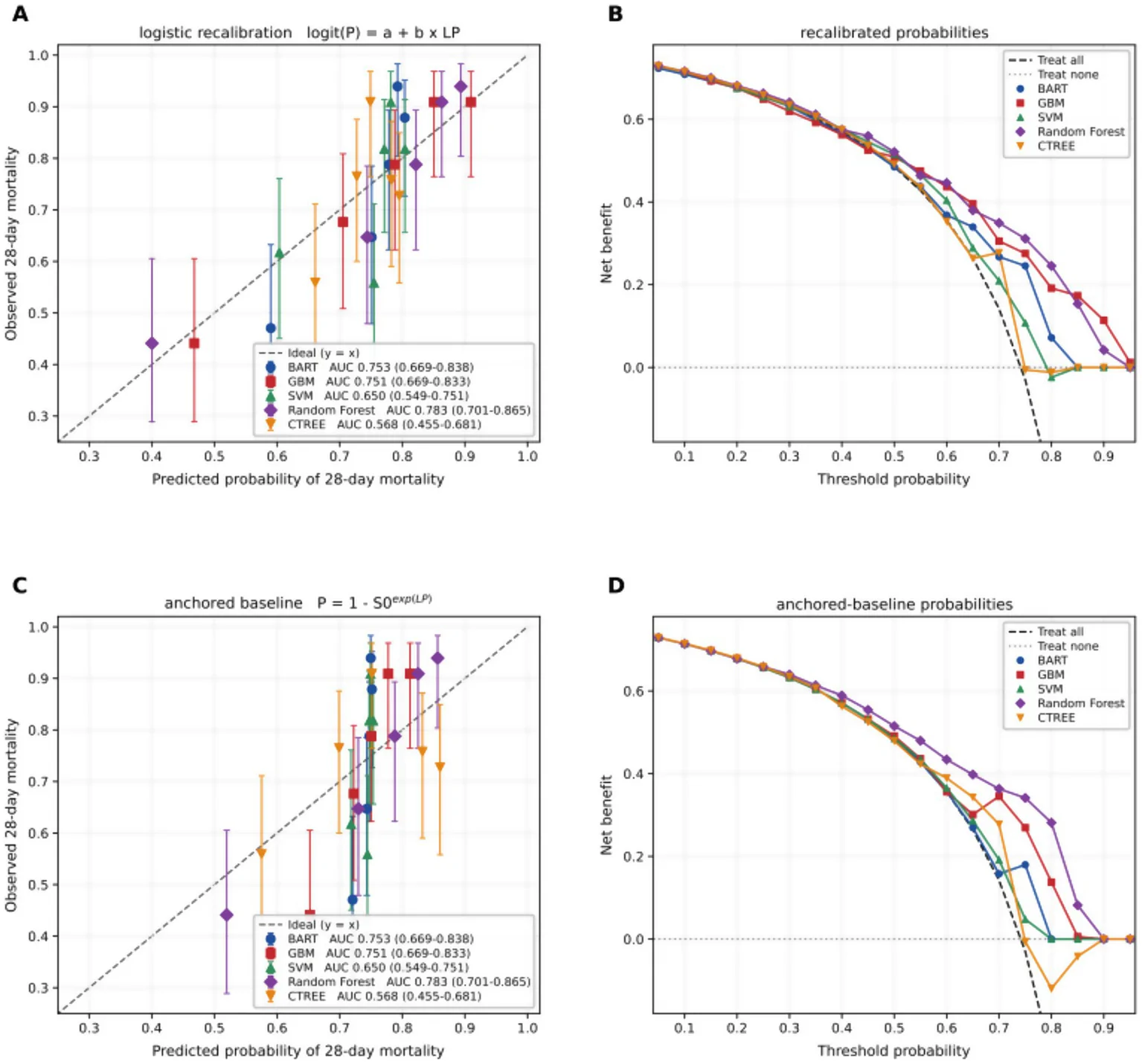
Calibration curves and decision curve analysis of five machine learning models. **(A)** Logistic recalibration calibration curves constructed by quintile stratification showing agreement between predicted and observed 28-day mortality rates. **(B)** Decision curve analysis based on recalibrated probabilities showing net benefit across clinically relevant threshold probabilities. **(C)** Anchored-baseline calibration curves (*p* = 1 − S0^exp.(LP)) showing agreement between predicted and observed 28-day mortality rates. **(D)** Decision curve analysis based on anchored-baseline probabilities. The models were trained using the 13 LASSO-selected variables. Area under the curve (AUC) values: Random Forest, 0.783; BART, 0.753; GBM, 0.751; SVM, 0.650; CTREE, 0.568. The dashed diagonal line represents a random classifier (AUC = 0.5). The Random Forest model achieved the best overall calibration-discrimination performance (Brier score = 0.155).

Decision curve analysis revealed that all five models provided greater clinical net benefit than either the “treat-all” or “treat-none” strategies across clinically relevant threshold probabilities (approximately 0.10–0.75; Figure 8). The Random Forest and GBM models consistently achieved the highest net benefit across the majority of the threshold range. At a threshold probability of 0.50, the net benefits were 0.521 (RF), 0.509 (GBM), 0.515 (SVM), 0.491 (CTREE), and 0.485 (BART), compared with 0.485 for the “treat-all” strategy. Notably, the Random Forest model maintained a positive net benefit even at higher threshold probabilities (≥0.75), whereas CTREE yielded negative net benefit at thresholds ≥0.75, suggesting potential misclassification of high-risk patients at extreme thresholds. These findings collectively support the clinical utility of the proposed models, particularly Random Forest and GBM, for risk stratification and clinical decision-making in TBI patients.

## Discussion

### Key statistical findings and their clinical relevance

This study of 837 ICU-admitted TBI patients demonstrates a significant nonlinear association between cardiac troponin T (cTnT) levels and 28-day all-cause mortality, with a potential risk threshold at cTnT ≥0.06 ng/mL. Using restricted cubic spline regression with three knots placed at 0.01, 0.04, and 0.11 ng/mL (selected based on the distribution of cTnT), we confirmed that the hazard ratio for mortality rose sharply below 0.1 ng/mL and continued to increase at higher concentrations. In multivariable Cox regression adjusted for 12 LASSO-selected covariates (excluding cTnT quartile itself), patients in Q3 (HR = 2.01, 95% CI: 1.37–2.95) and Q4 (HR = 1.96, 95% CI: 1.31–2.93) had nearly twofold higher mortality risks compared with Q1, while Q2 showed no significant difference. For predictive modeling, the Random Forest model achieved the highest discriminative performance (internal AUC = 0.783) and maintained moderate generalizability in external validation (AUC = 0.706; Figure 9). The model demonstrated good calibration (Brier score = 0.155) and favorable clinical utility across clinically relevant threshold probabilities as shown by decision curve analysis. Taken together, these findings confirm that cTnT is independently associated with 28-day mortality in TBI patients and provide a clinically interpretable machine learning model integrating cardiac biomarker information with traditional predictors.

**Figure 9:**
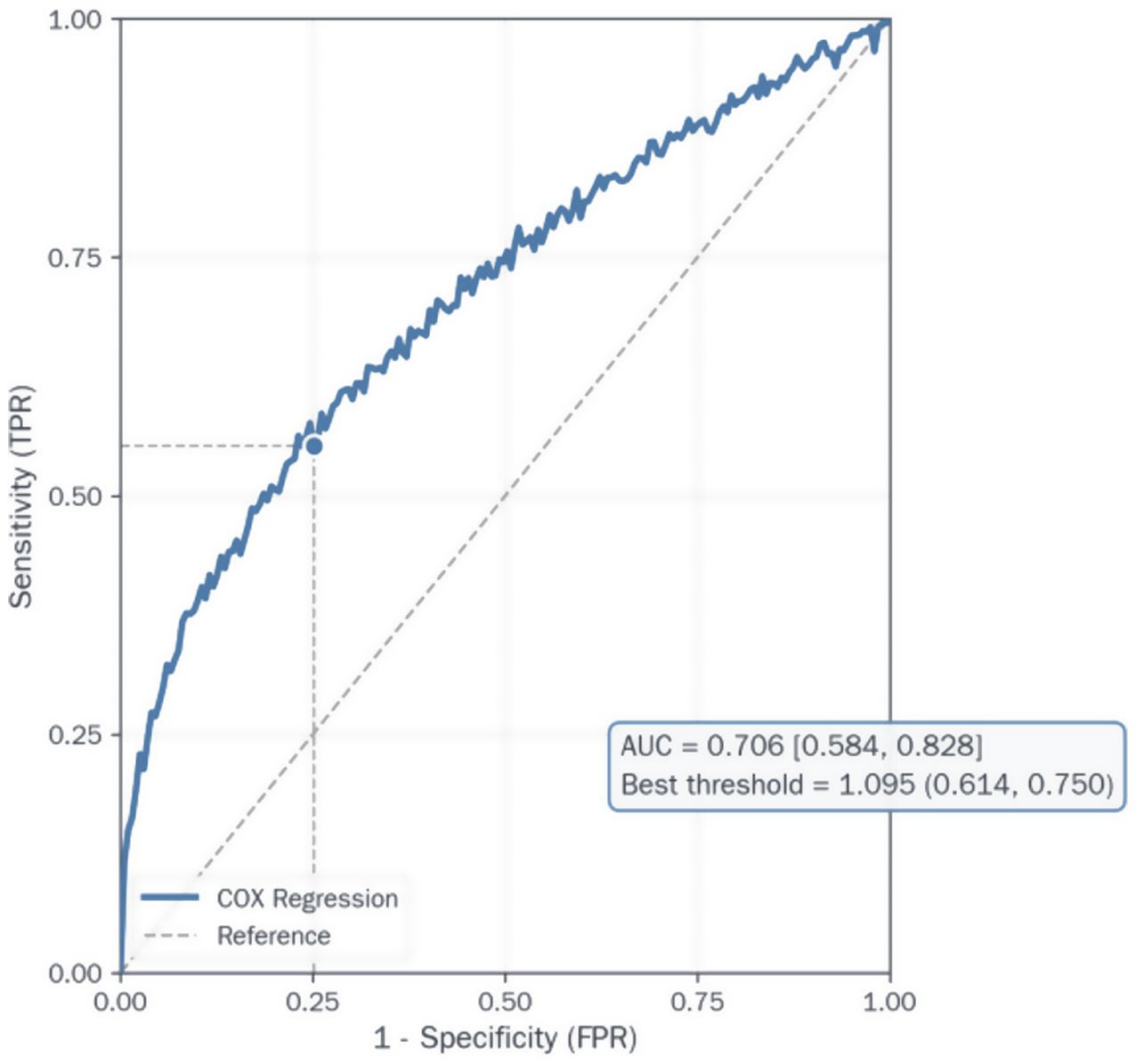
Receiver operating characteristic (ROC) curve of the Random Forest model for predicting 28-day all-cause mortality in the external single-center validation cohort (*n* = 121). The area under the curve (AUC) is 0.706 (95% CI: 0.584–0.828). The optimal threshold is 1.095, corresponding to a sensitivity of 0.614 and specificity of 0.750. The dashed diagonal line represents the reference of no discrimination (AUC = 0.5). The model was trained using the 13 LASSO-selected variables.

### Biological plausibility: cTnT as a marker of systemic critical illness

The strong association between cTnT and mortality may be explained by several interwoven pathophysiological mechanisms. First, elevated cTnT directly reflects myocardial injury (35–37) triggered by the sympathetic storm following TBI, which causes catecholamine-mediated cardiomyocyte damage even in the absence of coronary artery disease (38–41). Second, cTnT serves as a surrogate marker for systemic inflammatory severity (15, 42, 43); following TBI, damage-associated molecular patterns initiate cascades that damage cardiomyocytes through inflammatory cytokines and nitric oxide synthesis (44), a mechanism supported by our observation of concurrent leukocytosis and hyperglycemia in patients with elevated cTnT. Third, rising cTnT signifies multi-organ dysfunction, with parallel deterioration of liver and renal markers indicating that cTnT elevation is part of a systemic pathophysiological process rather than an isolated cardiac event (15, 42).

Notably, the cTnT–acute kidney injury (AKI) association carries distinct mechanistic implications at the cardio-renal interface. A bidirectional relationship exists: AKI may elevate cTnT through reduced renal clearance of troponin fragments (45, 46), while myocardial dysfunction can exacerbate renal hypoperfusion. This creates a “cardio-renal stress signature” in which injury to each organ amplifies the other through shared inflammatory and neurohormonal pathways (47, 48). This perspective reinforces cTnT as an integrative marker of systemic critical illness rather than a cardiac-specific signal, consistent with emerging evidence that troponin elevations in critically ill patients often signify multi-organ involvement (22, 23, 49, 50), with AKI-specific prognostic data further supporting this association (51).

### Methodological considerations: time windows and biomarker selection

Our study used the first cTnT measurement within 24 h of ICU admission, a window selected for clinical practicality. However, the choice of prediction time window may influence model performance. Prisco et al. (52) demonstrated that early metabolic derangements within 24 h predict TBI mortality, and Liu-DeRyke et al. (53) found first-day peak glucose levels superior to subsequent measurements, suggesting the first 24 h represent a critical prognostic window. Similarly, Hu et al. (54) reported that 24-h NEWS2 scores outperformed GCS in predicting TBI mortality. These studies collectively suggest that different time windows capture distinct pathophysiological processes: early markers such as hyperglycemia reflect acute stress responses, whereas cTnT—as a marker of myocardial injury and sustained systemic stress—may take longer to reach its prognostic peak. Future studies should explore multi-time-window, multi-marker strategies to improve risk stratification.

Sex differences in TBI outcomes have garnered increasing attention. A comprehensive scoping review by Gupte et al. found that human studies generally report worse outcomes in women compared with men, whereas animal studies often report better outcomes in females—a discrepancy likely attributable to interactions between injury severity, sample size, and experimental models (55). In our cohort, no significant sex difference was observed in mortality outcomes or across cTnT quartiles, which may be partly due to the predominance of male patients (63.80%) and the retrospective nature of the study. Nonetheless, the absence of a sex interaction in our analysis suggests that cTnT’s prognostic utility is robust across sexes, though this finding warrants prospective validation.

Our decision to focus on cTnT rather than troponin I (cTnI) was driven by data completeness in the MIMIC development cohort and by cTnT’s partial renal clearance (56), which renders it a more sensitive indicator of cardio-renal crosstalk—a key theme of our study. While growing evidence supports cTnI’s prognostic value in TBI (57–61), including a recently proposed cTnI/albumin index (59, 62), direct comparative studies in TBI populations are warranted. Additionally, the development and external cohorts used different cTnT assay platforms; although both are conventional-sensitivity assays with comparable detection limits, minor systematic differences may exist and should be considered when interpreting cross-cohort results.

### Paradoxical findings: vasopressor use and comorbidity patterns

A counterintuitive finding was the inverse association between vasopressor use and in-hospital mortality (HR = 0.507, 95% CI: 0.281–0.912), contrasting with its positive association with 28-day mortality (HR = 1.394). This discrepancy is most plausibly explained by time-dependent bias and confounding by indication: patients with catastrophic early mortality may die before vasopressor therapy is initiated, leading to misclassification as non-users (immortal time bias), while clinicians may preferentially administer vasopressors to patients perceived as having reversible hemodynamic states. This finding underscores the limitations of retrospective designs in capturing the timing of critical care interventions and should not be interpreted as evidence of a protective vasopressor effect.

Contrary to expectations, patients in the Q4 group had the lowest prevalence of hypertension, and the mortality group had lower hyperlipidemia prevalence—findings that appear to contradict studies reporting associations between hypertension and TBI outcomes (63, 64). We propose two explanations. First, patients with chronic hypertension may be protected via 20-HETE-mediated enhancement of myogenic constriction (65), and younger patients may mount more intense stress responses leading to greater myocardial injury. Second, elderly patients on long-term cardiovascular medications may benefit from “pharmacological pre-conditioning”; beta-blocker use may mitigate catecholamine excess (66–68), and statin therapy may attenuate inflammatory responses during acute injury. Although these findings require prospective validation, they suggest that underlying diseases and medication history modify the prognostic significance of cTnT.

### Study population specificity and age–cTnT interaction

Our cohort exhibited a notably high median GCS score (14 in both survivors and non-survivors), suggesting that the prognostic power of our model does not primarily rely on identifying patients with severe primary brain injury. Instead, its core value lies in discriminating high-risk individuals within a “complex” population with overlapping GCS scores, as signaled by elevated cTnT. The model maintained moderate discrimination (AUC = 0.706) in the external validation cohort with lower GCS, supporting its generalizability across TBI severities.

We also uncovered a striking age distribution pattern: the Q4 group (highest cTnT) had a significantly lower median age (68 years) than all other groups. We hypothesize two distinct pathophysiological processes. In elderly patients, mortality risk stems from age-related decline in physiological reserve and frailty; even mild injuries may lead to poor outcomes (69–72). Depreitere et al. (73) comprehensively reviewed the unique challenges in the assessment and management of TBI in older adults, emphasizing that this “silver trauma” poses distinct challenges to treating teams, including under-triage due to deceptively high GCS scores despite severe intracranial injury, the impact of frailty and comorbidities on clinical trajectories, and the need to avoid unsubstantiated nihilism. Notably, cTnT values are known to increase with age, with levels in individuals over 70 years potentially exceeding those in younger individuals by more than twofold (74–79). This age-related elevation may confound the interpretation of cTnT thresholds in elderly patients, as our identified threshold of ≥0.06 ng/mL may not be directly applicable to older adults without age adjustment. In contrast, markedly elevated cTnT in younger patients may signify an exaggerated stress response—a “catecholamine storm” triggering explosive myocardial injury and multi-organ failure. This is supported by our data showing the Q4 group had higher white blood cell counts, greater AKI incidence, more severe glucose disturbances, and the highest vasopressor use rate (60.68%). Age-related elevation of cTnT itself has been documented (74–79), and frailty may impose a “ceiling effect” on cTnT’s incremental prognostic value in the elderly, as suggested by recent work on frailty in TBI (80, 81). Nevertheless, subgroup analyses confirmed that cTnT’s prognostic value remained robust across all prespecified subgroups (all P for interaction > 0.05; Figure 5), including patients with heart failure and AKI.

### Clinical implications and limitations

Our findings suggest that cTnT ≥0.06 ng/mL may help identify TBI patients at higher risk, independent of GCS score. The model’s good calibration and favorable decision curve analysis support its potential as a clinical decision-support tool. However, given the moderate AUC in external validation (0.706) and limited sample size (*n* = 121), this threshold should be regarded as exploratory. The conventional-sensitivity assay used herein has a limit of detection of 0.01 ng/mL; this threshold is not directly interchangeable with high-sensitivity assays (where lower threshold values have been proposed) (82), and our results identify moderate-to-severe myocardial injury rather than minor stress. Future large-scale, multi-center prospective studies are needed to validate these findings and to determine whether age-specific cTnT thresholds or frailty assessment tools might further refine risk stratification in elderly TBI patients.

Several limitations should be noted. First, key variables including pH, ionized calcium, CRP, albumin, chronic kidney disease (83), and Injury Severity Score were unavailable. Second, we analyzed only the first cTnT measurement within 24 h, precluding assessment of serial changes; lack of echocardiography prevented determination of injury mechanism. Third, the development and external cohorts used different cTnT assay platforms, though both are conventional-sensitivity methods. Fourth, the retrospective design could not fully exclude unmeasured confounders. Detailed treatment protocols were not uniformly available in the MIMIC database, which may limit the comparability of management strategies between cohorts. Fifth, k-NN imputation of variables with <40% missingness may introduce residual bias. Sixth, requiring cTnT measurement may have excluded milder cases and very early deaths, potentially overestimating predictive performance. Finally, our single-center external validation cohort differed substantially from the development cohort in demographics and cTnT levels (Supplementary Table S9), warranting multi-center validation with standardized assays. Due to limited sample size of external validation cohort, we did not perform ablation analysis in the current work; this represents an important direction for future research. An ablation study quantifying cTnT’s incremental prognostic value would further strengthen our findings.

## Conclusion

This study demonstrates that cTnT is independently associated with 28-day mortality in TBI patients. Its prognostic value appears to be independent of the traditional GCS score. Notably, our data suggest that a cTnT level ≥ 0.06 ng/mL may help identify a subgroup of patients with higher systemic pathological burden, even when neurological function seems stable. However, given the moderate discriminative ability in the external validation cohort (AUC = 0.706) and the limited sample size of this cohort (*n* = 121), this threshold should be considered exploratory at present. If confirmed in future prospective, multi-center studies, cTnT could complement neurological assessment in a more integrated approach to TBI management, rather than replacing it.

## Data Availability

The datasets generated and/or analysed during the current study are available from the following sources: The development cohort data are from the publicly available MIMIC-III and MIMIC-IV databases (https://mimic.mit.edu/). The external validation cohort data from Taihe Hospital are not publicly available due to patient privacy restrictions but are available from the corresponding author on reasonable request and with permission from the Ethics Committee.
