# Supplementary material for: Cardiac troponin T and 28-day mortality in traumatic brain injury: a retrospective cohort study and machine learning prediction model
Source: Front Neurol. 2026 Oct 7;17:1926632. doi: 10.3389/fneur.2026.1926632 (PMC13683164; doi:10.3389/fneur.2026.1926632)
Supplement: Supplementary file 1 [file Supplementary_file_1.DOCX]

**Supplementary Table S1. Missing data proportions for candidate predictor variables before imputation (N=837)**

| **Variable** | **Overall (N=837)** | **Missing (%)** |
| --- | --- | --- |
| Demographics |  |  |
| Age, years | 75 (62–85) | 0.0 |
| Weight, kg | 75.5 (63.2–88.4) | 6.1 |
| Vital signs |  |  |
| Heart rate, beats/min | 88 (75–103) | 17.2 |
| Systolic blood pressure, mmHg | 127 (110–146) | 18.5 |
| Diastolic blood pressure, mmHg | 69 (59–81) | 18.5 |
| Mean arterial pressure, mmHg | 84.5 (74–97.8) | 18.5 |
| Respiratory rate, breaths/min | 19 (15–23) | 17.3 |
| SpO₂, % | 98 (95–100) | 17.3 |
| Neurological |  |  |
| Glasgow Coma Scale score | 14 (13–15) | 0.7 |
| Hematology |  |  |
| Hemoglobin, g/dL | 10.7 (9.3–12.3) | 0.7 |
| Platelet count, ×10⁹/L | 182 (138–236.8) | 0.8 |
| White blood cell count, ×10⁹/L | 10.6 (7.6–14.2) | 0.8 |
| Biochemistry |  |  |
| Albumin, g/dL | 3.2 (2.7–3.6) | 33.3 |
| Calcium, mg/dL | 8.45 (7.8–9.0) | 0.4 |
| Chloride, mmol/L | 104 (101–108) | 0.2 |
| Bedside glucose, mg/dL | 138 (111–175.5) | 0.2 |
| Potassium, mmol/L | 4.1 (3.7–4.6) | 0.2 |
| Sodium, mmol/L | 140 (137–142) | 0.2 |

*Note: All variables listed above are presented in Table 1 of the main manuscript. Missing rates were all below the pre-specified 40% exclusion threshold. Imputation was performed using k-nearest neighbors (k-NN) with k=5 selected by cross-validation. Categorical variables (sex, comorbidities, vasopressor use) had no missing values.*

**Supplementary Table S2. Univariate Cox regression analysis of cTnT quartiles and mortality**

| **Outcome** | **cTnT group** | **HR (95% CI)** | **P value** |
| --- | --- | --- | --- |
| 28-day mortality | Q2 vs. Q1 | 0.978 (0.616–1.554) | 0.925 |
|  | Q3 vs. Q1 | 2.002 (1.385–2.895) | <0.001 |
|  | Q4 vs. Q1 | 2.000 (1.382–2.895) | <0.001 |
| In-hospital mortality | Q2 vs. Q1 | 1.149 (0.763–1.730) | 0.506 |
|  | Q3 vs. Q1 | 1.816 (1.281–2.575) | 0.001 |
|  | Q4 vs. Q1 | 1.862 (1.315–2.637) | <0.001 |

*Note: Unadjusted models, no covariates included.*

**Supplementary Table S3. Hyperparameters and internal performance metrics of the final Random Forest model**

| **Parameter** | **Symbol** | **Value** | **Description** |
| --- | --- | --- | --- |
| Maximum tree depth | max.depth | 30 | Max depth limit for individual trees to prevent overfitting |
| Minimum samples at leaf | min.bucket | 2 | Minimum samples required at a leaf node |
| Minimum samples at split | min.node.size | 4 | Minimum samples required to split a node |
| Number of trees | num.trees | 600 | Total number of decision trees in the forest |
| Number of features to sample | mtry | 3 | Features randomly sampled at each split |

**Supplementary Table S4. Boruta variable importance analysis**

| **Variable** | **mean Imp** | **median Imp** | **min Imp** | **max Imp** | **norm Hits** | **decision** |
| --- | --- | --- | --- | --- | --- | --- |
| Lactate | 15.188398868885 | 15.1373010779293 | 12.9474378262042 | 17.6674475992876 | 1 | Confirmed |
| White blood cell count | 13.3044043152425 | 13.3718683185746 | 10.2606243566659 | 15.6104601264729 | 1 | Confirmed |
| PaCO₂ | 10.4882408959476 | 10.3771651153938 | 7.93061752225409 | 13.5727398413927 | 1 | Confirmed |
| Bedside glucose | 10.2810034404697 | 10.2313250662939 | 7.70118564111599 | 12.1163665761609 | 1 | Confirmed |
| Vasopressor use | 7.65889537625596 | 7.61477496917419 | 5.68446395734709 | 9.3587247404056 | 1 | Confirmed |
| Glasgow Coma Scale score | 7.64558735417358 | 7.69753495313902 | 4.77215849179038 | 9.67935118189731 | 1 | Confirmed |
| Calcium | 7.63895344670082 | 7.57834140710308 | 5.44344677605295 | 9.4577534103678 | 1 | Confirmed |
| PaO₂ | 6.22324121766722 | 6.34315405076175 | 3.52759323938753 | 8.1048174263121 | 0.97979797979798 | Confirmed |
| Cardiac troponin T quartile | 5.71726915683005 | 5.77618589242645 | 3.38695662735141 | 7.39993596739407 | 0.98989898989899 | Confirmed |
| Age | 5.41825871610974 | 5.36738656566463 | 2.50681135827388 | 8.40575806513339 | 0.96969696969697 | Confirmed |
| Aspartate aminotransferase | 5.30487680218174 | 5.39314247794004 | 2.83180512244579 | 8.13938940973752 | 0.95959595959596 | Confirmed |
| Total bilirubin | 4.96374644956063 | 4.97846235200335 | 2.6944931635589 | 7.60960448262831 | 0.949494949494949 | Confirmed |
| Heart rate | 4.14922161552663 | 4.12099029242365 | 1.82708845917673 | 7.00994444691789 | 0.848484848484848 | Confirmed |
| Platelet count | 3.95160687946684 | 3.86575239926139 | 1.25237633648239 | 5.87232448681971 | 0.858585858585859 | Confirmed |
| Mean arterial pressure | 3.82052527271349 | 3.91411275097417 | 1.64954776531126 | 5.85718564864036 | 0.848484848484848 | Confirmed |
| Sodium | 3.79297500022712 | 3.76787659327505 | 0.838571013247158 | 7.27851511793348 | 0.868686868686869 | Confirmed |
| Systolic blood pressure | 3.47394040398442 | 3.51784078855727 | 0.333010875224326 | 5.73025849857176 | 0.747474747474748 | Confirmed |
| Hemoglobin | 3.27453198913605 | 3.1954086044492 | 0.779854403713639 | 6.31568720798889 | 0.737373737373737 | Confirmed |
| Blood urea nitrogen | 3.27215429187317 | 3.10049245282025 | 0.934793674004948 | 5.65076632957199 | 0.727272727272727 | Confirmed |
| Respiratory rate | 3.13088914407548 | 3.13418361624937 | 0.301381054720476 | 5.31083860233349 | 0.696969696969697 | Confirmed |
| Creatinine | 3.04551461706153 | 3.18957952671258 | 0.883509971259551 | 4.62741897601098 | 0.717171717171717 | Confirmed |
| Alanine aminotransferase | 3.04237465864559 | 3.118530166966 | 0.709381544827425 | 5.32083395756558 | 0.676767676767677 | Confirmed |
| Diastolic blood pressure | 2.85846257627328 | 2.84442509292389 | -0.0134374805843363 | 5.62062328345746 | 0.575757575757576 | Tentative |
| Potassium | 2.03808075813408 | 1.9998163668079 | 0.0581713070660941 | 5.32178837079541 | 0.242424242424242 | Rejected |
| Sex | 1.29839928490287 | 1.17433861672211 | -0.125471888371209 | 3.08426218915553 | 0.0101010101010101 | Rejected |
| Hyperlipidemia | 1.07166074416581 | 1.35833340065442 | -0.963623896687365 | 2.59493688969114 | 0.0101010101010101 | Rejected |
| Acute kidney injury | 1.03147464709081 | 0.874010448645451 | -0.880849314221117 | 3.12140263313632 | 0.0101010101010101 | Rejected |
| Heart failure | 1.02841404372322 | 1.1870003677351 | -2.24095773685961 | 2.43182308456524 | 0.0202020202020202 | Rejected |
| Cerebrovascular disease | 0.968967684354195 | 1.08098588263669 | -0.455769890002112 | 2.15386349507051 | 0 | Rejected |
| Type 2 diabetes | 0.639337309337412 | 0.533219001279125 | -0.751473137390725 | 3.06026411952489 | 0.0303030303030303 | Rejected |
| Hypertension | 0.513297314606276 | 0.511536941645994 | -1.33867994710533 | 3.46188994748801 | 0.0101010101010101 | Rejected |

**Supplementary Table S5. Multivariable Cox regression analysis of cTnT quartiles and 28-day all-cause mortality**

| **Characteristics** | **Number(%)** | **Hazard Ratio (HR)** | **Lower 95%** | **Upper 95%** | **P value** |
| --- | --- | --- | --- | --- | --- |
| Age | 70.96 (17.79) | 1.021 | 1.012 | 1.03 | 0 |
| Aspartate aminotransferase | 156.50 (437.62) | 1 | 1 | 1 | 0.242 |
| Creatinine | 1.63 (1.68) | 0.947 | 0.859 | 1.043 | 0.269 |
| Glasgow Coma Scale score | 12.95 (3.11) | 0.945 | 0.909 | 0.983 | 0.005 |
| Bedside glucose | 162.00 (106.54) | 1.002 | 1.001 | 1.003 | 0.001 |
| Hemoglobin | 10.82 (2.23) | 1.041 | 0.982 | 1.104 | 0.175 |
| Lactate | 2.76 (2.01) | 1.114 | 1.054 | 1.178 | 0 |
| PaO₂ | 150.76 (98.88) | 1.001 | 1 | 1.002 | 0.109 |
| Sodium | 139.32 (5.47) | 1.028 | 1.003 | 1.054 | 0.025 |
| Total bilirubin | 0.97 (1.41) | 1.108 | 1.048 | 1.172 | 0 |
| Cardiac troponin T quartile |  |  |  |  |  |
| 1 | 265 (31.7) |  |  |  |  |
| 2 | 159 (19.0) | 1.124 | 0.703 | 1.797 | 0.626 |
| 3 | 207 (24.7) | 2.009 | 1.369 | 2.948 | 0 |
| 4 | 206 (24.6) | 1.956 | 1.307 | 2.927 | 0.001 |
| Vasopressor use |  |  |  |  |  |
| 0 | 470 (56.2) |  |  |  |  |
| 1 | 367 (43.8) | 1.394 | 1.032 | 1.881 | 0.03 |
| White blood cell count | 11.78 (6.93) | 1.033 | 1.017 | 1.048 | 0 |

**Supplementary Table S6. Multivariable Cox regression analysis of cTnT quartiles and in-hospital all-cause mortality**

| **Characteristics** | **Number(%)** | **Hazard Ratio (HR)** | **Lower 95%** | **Upper 95%** | **P value** |
| --- | --- | --- | --- | --- | --- |
| Age | 70.96 (17.79) | 1.058 | 1.033 | 1.083 | 0 |
| Aspartate aminotransferase | 156.50 (437.62) | 1.001 | 1 | 1.001 | 0.011 |
| Creatinine | 1.63 (1.68) | 0.958 | 0.805 | 1.139 | 0.626 |
| Glasgow Coma Scale score | 12.95 (3.11) | 0.883 | 0.819 | 0.953 | 0.001 |
| Bedside glucose | 162.00 (106.54) | 1.003 | 1.001 | 1.005 | 0.001 |
| Hemoglobin | 10.82 (2.23) | 1.034 | 0.911 | 1.174 | 0.601 |
| Lactate | 2.76 (2.01) | 0.966 | 0.815 | 1.146 | 0.691 |
| PaO₂ | 150.76 (98.88) | 1 | 0.997 | 1.003 | 0.931 |
| Sodium | 139.32 (5.47) | 1.046 | 1.01 | 1.083 | 0.012 |
| Total bilirubin | 0.97 (1.41) | 1.116 | 0.966 | 1.289 | 0.137 |
| Cardiac troponin T quartile |  |  |  |  |  |
| 1 | 265 (31.7) |  |  |  |  |
| 2 | 159 (19.0) | 2.043 | 0.895 | 4.666 | 0.09 |
| 3 | 207 (24.7) | 2.921 | 1.424 | 5.992 | 0.003 |
| 4 | 206 (24.6) | 2.495 | 1.084 | 5.747 | 0.032 |
| Vasopressor use |  |  |  |  |  |
| 0 | 470 (56.2) |  |  |  |  |
| 1 | 367 (43.8) | 0.507 | 0.281 | 0.912 | 0.023 |
| White blood cell count | 11.78 (6.93) | 1.05 | 1.025 | 1.075 | 0 |

**Supplementary Table S7. LASSO regression coefficients for all candidate predictors**

| **Variable** | **Coefficient (lambda.min)** |
| --- | --- |
| Age | 0.0114639750764333 |
| Heart rate | 0 |
| Systolic blood pressure | 0 |
| Mean arterial pressure | 0 |
| Respiratory rate | 0 |
| Glasgow Coma Scale score | -0.0329951592361104 |
| Hemoglobin | 0.00907913621303657 |
| Platelet count | 0 |
| White blood cell count | 0.0301390563125318 |
| Calcium | 0 |
| Bedside glucose | 0.00119510314907651 |
| Sodium | 0.0104393027258978 |
| Lactate | 0.106812557458404 |
| PaCO₂ | 0 |
| PaO₂ | 0.000237590299432469 |
| Alanine aminotransferase | 0 |
| Aspartate aminotransferase | 9.8255001021635e-05 |
| Total bilirubin | 0.0596402052353646 |
| Creatinine | -0.00564935330734619 |
| Blood urea nitrogen | 0 |
| Cardiac troponin T quartile | 0.160487977153597 |
| Vasopressor use | 0.125848464142642 |

**Supplementary Table S8. Model performance comparison: discrimination, calibration, and overall accuracy**

| **Model** | **AUC (95% CI)** | **Calibration slope b** | **Brier (calibration)** | **Brier (recalibration)** | **Scaled Brier** |
| --- | --- | --- | --- | --- | --- |
| Random Forest | 0.783 (0.701–0.865) | 2.67 | 0.1569 | 0.1545 | 19.2% |
| BART | 0.753 (0.669–0.838) | 12.86 | 0.1878 | 0.1780 | 6.9% |
| GBM | 0.751 (0.669–0.833) | 5.47 | 0.1751 | 0.1647 | 13.9% |
| SVM | 0.650 (0.549–0.751) | 10.86 | 0.1870 | 0.1787 | 6.5% |
| CTREE | 0.568 (0.455–0.681) | 0.75 | 0.1886 | 0.1858 | 2.8% |
| Null model | 0.500 | — | 0.1912 | 0% |  |

**Supplementary Table S9. Baseline characteristics of the development (MIMIC-III/IV) and external validation (Taihe Hospital) cohorts**

| **Variable** | **MIMIC (N = 837)** | **Taihe Hospital (N = 121)** | **P value** |
| --- | --- | --- | --- |
| Demographics |  |  |  |
| Age, years | 75.00 (62.00–85.00) | 57 (47–67) | <0.001 |
| Sex, n (%) | 534 (63.80) | 82 (67.77) | 0.418 |
| Weight, kg | 76.00 (64.40–88.00) | 80 (70–97) | <0.001 |
| Vital signs |  |  |  |
| Heart rate, beats/min | 91.00 (78.00–101.50) | 84 (76–97) | 0.012 |
| Systolic blood pressure, mmHg | 131.90 (115.00–145.00) | 137 (123–156) | 0.002 |
| Diastolic blood pressure, mmHg | 71.00 (61.00–80.00) | 80 (72–89) | <0.001 |
| Mean arterial pressure, mmHg | 86.00 (77.00–95.60) | 98.67 (92.00–107.67) | <0.001 |
| Respiratory rate, breaths/min | 19.00 (16.00–22.00) | 16 (15–20) | <0.001 |
| SpO₂, % | 97.00 (94.00–100.00) | 97 (94–100) | 0.898 |
| Neurological status |  |  |  |
| Glasgow Coma Scale score | 14.00 (13.00–15.00) | 6 (4–12) | <0.001 |
| Laboratory parameters |  |  |  |
| Hemoglobin, g/dL | 10.70 (9.30–12.30) | 12.60 (10.90–14.10) | <0.001 |
| Platelet count, ×10⁹/L | 182.00 (138.00–236.00) | 178 (149–230) | 0.937 |
| White blood cell count, ×10⁹/L | 10.60 (7.60–14.10) | 15.07 (11.59–19.81) | <0.001 |
| Albumin, g/dL | 3.11 (2.81–3.40) | 3.03 (2.70–3.50) | 0.276 |
| Calcium, mg/dL | 8.40 (7.80–9.00) | 7.92 (7.60–8.16) | <0.001 |
| Chloride, mmol/L | 104.00 (101.00–108.00) | 107.70 (105.80–110.40) | <0.001 |
| Glucose, mg/dL | 138.00 (111.00–175.00) | 156.24 (133.74–206.46) | <0.001 |
| Potassium, mmol/L | 4.10 (3.70–4.60) | 3.87 (3.62–4.13) | <0.001 |
| Sodium, mmol/L | 140.00 (137.00–142.00) | 138.40 (136.50–140.40) | 0.001 |
| Bicarbonate, mmol/L | 23.40 (21.00–26.00) | 22 (20–24) | <0.001 |
| Lactate, mmol/L | 2.30 (1.50–3.20) | 2.50 (1.60–4.00) | 0.045 |
| PaCO₂, mmHg | 40.20 (36.00–46.00) | 39.30 (35.00–42.50) | 0.004 |
| PaO₂, mmHg | 124.30 (79.00–195.00) | 145.60 (99.00–194.50) | 0.018 |
| Liver and renal function |  |  |  |
| Alanine aminotransferase, U/L | 45.90 (23.00–92.00) | 23.20 (15.70–36.80) | <0.001 |
| Aspartate aminotransferase, U/L | 73.00 (37.00–141.00) | 36.90 (28.00–58.20) | <0.001 |
| Total bilirubin, mg/dL | 0.75 (0.50–1.03) | 0.78 (0.57–1.04) | 0.221 |
| Creatinine, mg/dL | 1.10 (0.80–1.60) | 0.73 (0.58–0.85) | <0.001 |
| Blood urea nitrogen, mg/dL | 22.00 (15.00–35.00) | 14.39 (11.73–17.84) | <0.001 |
| Cardiac injury marker |  |  |  |
| Troponin T, ng/mL | 0.06 (0.03–0.17) | 0.03 (0.02–0.07) | <0.001 |
| Comorbidities |  |  |  |
| Hypertension, n (%) | 334 (39.90) | 53 (43.80) | 0.429 |
| Acute kidney injury, n (%) | 265 (31.66) | 20 (16.53) | <0.001 |
| Cerebrovascular disease, n (%) | 74 (8.84) | 5 (4.13) | 0.109 |
| Type 2 diabetes, n (%) | 230 (27.48) | 29 (23.97) | 0.445 |
| Hyperlipidemia, n (%) | 217 (25.93) | 6 (4.96) | <0.001 |
| Heart failure, n (%) | 250 (29.87) | 32 (26.45) | 0.522 |
| Therapeutic interventions |  |  |  |
| Vasopressor use, n (%) | 367 (43.85) | 54 (44.63) | 0.922 |
| Outcomes |  |  |  |
| 28-day mortality, n (%) | 217 (25.93) | 21 (17.36) | 0.043 |
| Hospital length of stay, days | 9.65 (4.93–17.24) | 12.97 (5.68–22.98) | 0.014 |
| ICU length of stay, days | 3.08 (1.51–7.00) | 4.13 (1.88–10.79) | 0.014 |
